# Genome assembly of pomegranate highlights structural variations driving population differentiation and key loci underpinning cold adaption

**DOI:** 10.1093/hr/uhaf022

**Published:** 2025-01-21

**Authors:** Xiang Luo, Zhenyang Shua, Diguang Zhao, Beibei Liu, Hua Luo, Ying Chen, Dong Meng, Zhihua Song, Qing Yang, Zicheng Wang, Dong Tang, Xingguo Zhang, Juan Zhang, Kai Ma, Wen Yao

**Affiliations:** College of Agriculture, Henan University, No. 379 North Section of Mingli Road, Zhengdong New District, Zhengzhou 450046, Henan, China; Institute of Horticultural and Crops, Xinjiang Academy of Agricultural Sciences, No. 403 Nanchang Road, Urumqi 830013, Xinjiang, China; National Key Laboratory of Wheat and Maize Crop Science, College of Life Sciences, Henan Agricultural University, No. 218 Ping'an Avenue, Zhengdong New District, Zhengzhou 450046, Henan, China; Zhengzhou Fruit Research Institute, Chinese Academy of Agricultural Sciences, Southern End of Weilai Road, Guancheng District, Zhengzhou 450009, Henan, China; Zhengzhou Fruit Research Institute, Chinese Academy of Agricultural Sciences, Southern End of Weilai Road, Guancheng District, Zhengzhou 450009, Henan, China; Zaozhuang Pomegranate Research Institute, Zaozhuang Pomegranate National Forest Germplasm Resource Bank, Shiliu Avenue, Yicheng District, Zaozhuang 277300, Shandong, China; Zaozhuang Pomegranate Research Institute, Zaozhuang Pomegranate National Forest Germplasm Resource Bank, Shiliu Avenue, Yicheng District, Zaozhuang 277300, Shandong, China; College of Forestry, Beijing Forestry University, No. 35 Tsinghua East Road, Haidian District, Beijing 100083, China; College of Forestry, Beijing Forestry University, No. 35 Tsinghua East Road, Haidian District, Beijing 100083, China; College of Forestry, Beijing Forestry University, No. 35 Tsinghua East Road, Haidian District, Beijing 100083, China; College of Agriculture, Henan University, No. 379 North Section of Mingli Road, Zhengdong New District, Zhengzhou 450046, Henan, China; Bioyi Biotechnology Co., Ltd., No. 888 Gaoxin Avenue, East Lake High-Tech Development Zone, Wuhan 430075, Hubei, China; Bioyi Biotechnology Co., Ltd., No. 888 Gaoxin Avenue, East Lake High-Tech Development Zone, Wuhan 430075, Hubei, China; Research Institute of Biology and Agriculture, University of Science and Technology Beijing, No. 30 Xueyuan Road, Haidian District, Beijing 100083, China; Institute of Horticultural and Crops, Xinjiang Academy of Agricultural Sciences, No. 403 Nanchang Road, Urumqi 830013, Xinjiang, China; National Key Laboratory of Wheat and Maize Crop Science, College of Life Sciences, Henan Agricultural University, No. 218 Ping'an Avenue, Zhengdong New District, Zhengzhou 450046, Henan, China

## Abstract

Cold damage poses a significant challenge to the cultivation of soft-seeded pomegranate varieties, hindering the growth of the pomegranate industry. The genetic basis of cold tolerance in pomegranates has remained elusive, largely due to the lack of high-quality genome assemblies for cold-tolerant varieties and comprehensive population-scale genomic studies. In this study, we addressed these challenges by assembling a high-quality chromosome-level reference genome for 'Sanbai', a pomegranate variety renowned for its freezing resistance, achieving an impressive contig N50 of 15.93 Mb. This robust assembly, enhanced by long-read sequencing of 38 pomegranate accessions, facilitated the identification of 14 239 polymorphic structural variants, revealing their critical roles in genomic diversity and population differentiation related to cold tolerance. Of particular significance was the discovery of a ~ 5.4-Mb inversion on chromosome 1, which emerged as an important factor affecting cold tolerance in pomegranate. Moreover, through the integration of bulked segregant analysis, differential selection analysis, and genetic transformation techniques, we identified and validated the interaction between the PgNAC12 transcription factor and *PgCBF1*, disclosing their pivotal roles in response to cold stress. These findings mark a significant advancement in pomegranate genomics, offering novel insights into the genetic mechanisms of cold tolerance and providing valuable resources for the genetic improvement of soft-seeded pomegranate varieties.

## Introduction

Pomegranate (*Punica granatum* L, 2*n* = 16), a member of the Lythraceae family, is native to Central Asia, with Iran as a primary center of origin [1, 2]. Owning to the considerable nutritional, medicinal, pharmaceutical, and cosmeceutical benefits of its fruits [1], pomegranate has become a widely cultivated and economically important fruit tree around the world. Over time, both natural and human selection have contributed to a broad spectrum of phenotypic diversity in pomegranate. This diversity encompasses variations in vegetative growth, floral characteristics, phenology, fruit morphology, color, seed hardness, stress resistance, and even chromosome number. Pomegranate varieties with soft seeds are particularly valued for their appealing internal quality, resulting in increased consumer acceptance and elevated market prices. However, it is important to note that nearly all one-year-old soft-seeded pomegranate varieties are highly susceptible to cold damage [3, 4], with damage typically concentrated in the stem segments near the ground. Therefore, identifying genomic variations and candidate genes associated with cold tolerance has become a primary objective in current pomegranate breeding programs.

Advancements in high-throughput sequencing technologies have markedly improved the efficiency of genomic analyses, population genetic analyses, and gene mapping analyses, enabling a more comprehensive exploration of genomic variations and their biological functions. Previously, the genomes of two hard-seeded pomegranate accessions, 'Dabenzi' and 'Taishanhong', were assembled using short-read sequencing data [2, 5]. More recently, we reported the first high-quality genome assembly of a soft-seeded pomegranate accession, 'Tunisia', utilizing long-read sequencing technology [6]. These genome assemblies have enabled the identification of genetic variations associated with important quantitative traits in pomegranate, such as genes related to cell wall structure (*CCR*, *CAD*, *CelSy*, *SuSy*, *CCoA-OMT*) and transcription factors (MYB, NAC, WRKY, and MYC) implicated in seed hardness development [5–12]. Additionally, genes involved in the FoxO and MAPK signaling pathways have been identified as playing critical roles in the pomegranate's response to cold stress [6].

Despite these advancements, our understanding of the full spectrum of genomic variations and functional genes regulating cold stress tolerance in pomegranate remains incomplete. A significant limitation is the absence of a high-quality reference genome for hard-seeded pomegranate, which hampers our ability to comprehensively catalog genomic variations across pomegranate varieties. Furthermore, our knowledge regarding genomic variations, particularly large-scale structural variations (SVs) within pomegranate populations, is still limited. Although we have previously identified specific selected regions associated with cold tolerance [6], pinpointing functional genes remains challenging due to the lack of corroborative evidence.

In response to these challenges, a collaborative effort involving four institutions has been initiated with the following objectives: (i) to construct a chromosome-level reference genome for 'Sanbai', known for its cold tolerance and hard seeds, by integrating PacBio, Hi-C, and Illumina sequencing technologies; (ii) to comprehensively catalog genomic variations, particularly SVs, across the pomegranate population, elucidating their roles in genetic diversity, population differentiation, and gene discovery; and (iii) to perform selective sweep analysis, bulked segregant analysis (BSA), and genetic transformation to identify key genes regulating cold tolerance. The outcomes of this study represent the most extensive and comprehensive resource available for pomegranate to date, offering valuable insights for functional genomics studies and facilitating genomic-assisted improvement of pomegranate.

## Results

### High-quality *de novo* genome assembly of 'Sanbai'

**Figure 1:**
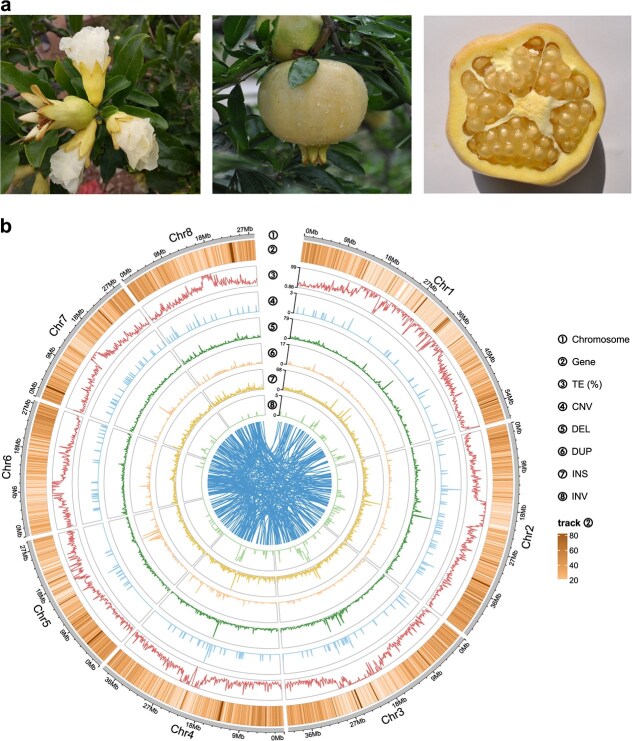
Representative phenotypes and genomic features of 'Sanbai'. (a) The flower, mature fruit, and mature seed of 'Sanbai'. (b) Genomic features of the high-quality assembly of 'Sanbai'. The outer-to-inner tracks of the Circos plot display the following: the eight chromosomes, gene number, TE (transposable element) density (%), CNV (copy number variation) number, DEL (deletion) number, DUP (duplication) number, INS (insertion) number, and INV (inversion) number, each plotted within 100-kb genomic regions of the 'Sanbai' genome assembly. The innermost part of the Circos plot displays syntenic blocks among chromosomes

'Sanbai' is a pomegranate variety recognized for its hard seeds, freezing resistance, and the lack of anthocyanins (Fig. 1a). The genome size of 'Sanbai' was estimated to be 310.83 Mb, with a heterozygosity rate of 0.3% (Fig. S1), based on a *K*-mer analysis of 98-fold Illumina sequencing data (30.55 Gb clean reads). To accurately profile the genomic divergence between soft- and hard-seeded pomegranates, we conducted a *de novo* genome assembly of 'Sanbai' by integrating multiple sequencing technologies, including PacBio long-read sequencing, Illumina short-read sequencing, and high-throughput chromosome conformation capture (Hi-C) sequencing (see section Materials and methods) (Fig. 1b).

We first utilized PacBio sequencing data with over 180-fold coverage (56.08 Gb clean data) to perform the initial contig assembly, resulting in 283 contigs (Table 1). Subsequently, we employed Illumina paired-end reads to correct and refine these contigs, achieving an assembly with 283 contigs and a total length of 318.59 Mb (Table 1). The size is closely aligned with the recently published 'Tunisia' genome (320.31 Mb) [6]. The N50 of our 283 contigs is 15.93 Mb, significantly longer than that of 'Tunisia' (contig N50: 4.49 Mb) [6], 'Dabenzi' (contig N50: 66.97 kb) [5], and 'Taishanhong' (contig N50: 97 kb) [2] (Table 1). To further enhance the assembly, we leveraged 32.68 Gb of Hi-C data (105-fold genome coverage) to anchor the 283 contigs onto eight chromosomes (2*n* = 16), resulting in 243 scaffolds with an N50 length of 39.26 Mb (Fig. S2, Table 1). The scaffold lengths ranged from a minimum of 1000 bp to a maximum of 58.06 Mb, with approximately 92.89% of the total assembled sequences successfully anchored onto the eight chromosomes. The overall GC content of the genome was calculated as 40.48%, which is comparable to that of 'Tunisia' (40.38%) [6].

Benchmarking Universal Single-Copy Orthologs (BUSCO) analysis recovered 97.18% of conserved single-copy eukaryotic genes, while CEGMA analysis identified 95.56% of highly complete core genes within the 'Sanbai' genome. Additionally, over 95.56% of the 'Sanbai' genome could be covered by PacBio sequencing reads from 'Tunisia'. To further assess the continuity of the 'Sanbai' assembly, we calculated the LTR Assembly Index (LAI), which evaluates the completeness of LTR retrotransposons [13]. The 'Sanbai' genome achieved a mean LAI score of 16.42, which is comparable to that of the 'Tunisia' genome (17.94) but significantly higher than that of 'Dabenzi' (0.10) and 'Taishanhong' (1.55) (Fig. S3). Moreover, our analysis revealed significant collinearity between the genome assemblies of 'Sanbai' and 'Tunisia' (Figs S4 and S5). These results collectively indicate the high quality of the 'Sanbai' genome assembly.

### Annotation of transposons and protein-coding genes in the genome of 'Sanbai'

Using RepeatMasker and the Repbase repeat library (v15.02), we identified a total of 366 987 transposable elements (TEs) in the 'Sanbai' genome (Table S1). These TEs collectively spanned 141.13 Mb, representing 45.40% of the genome. Among these TEs, 31.66% were long terminal repeat (LTR) retrotransposons, and 68.34% were DNA transposons. Notably, the LTR retrotransposons included 46 910 Gypsy elements and 18 225 Copia elements, covering 41.94 and 12.35 Mb of the genome, respectively. Among DNA transposons, Helitrons were the most abundant, with 175 765 elements spanning a total of 38.56 Mb.

The annotation of protein-coding genes was conducted by integrating ab initio prediction, homology-based protein mapping, RNA-seq data mapping, and PacBio IsoSeq transcript mapping (see section Materials and methods). In total, we annotated 32 319 protein-coding genes in the 'Sanbai' genome (Table 1), which is comparable to the 33 594 protein-coding genes annotated in the 'Tunisia' genome. Notably, 52.87% of these gene models were supported by full-length transcripts with coverage exceeding 50%, and 83.04% were supported by Illumina RNA-seq data with coverage >50% (Table S2). The average lengths of genes, mRNAs, exons, and introns were 2587.31, 1237, 259.42, and 348 bp, respectively (Table 1), which were close to those of the 'Tunisia' genome (Fig. S6). Additionally, we functionally annotated 98.11% of the genes using various databases, including KOG (43.44%), Kyoto Encyclopedia of Genes and Genome (KEGG, 29.20%), NCBI NR (97.22%), Swiss-Prot (63.83%), and Gene Ontology (GO, 70.29%) (Table S3).

**Table 1:** Characteristics of the 'Sanbai' genome assembly.

Assembly feature	Statistics
Assembly length	318.59 Mb
Chromosome number (2*n*)	2 × 8
Assembled sequences anchored to chromosomes (%)	92.15%
Number of contigs	283
Contig N50	15.93 Mb
Longest contig	38.05 Mb
Number of scaffolds	243
Scaffold N50	35.49 Mb
Proportion of transposons (%)	49.70%
Number of annotated gene models	31 667
Average gene length	2587.31 bp
Average intron length	348 bp
Average exon length	259.42 bp
Average mRNA length	1237 bp
GC content (%)	40.48%
Number of microRNA	125
Number of small nuclear RNA	261
Number of transfer RNA	718
Number of ribosomal RNA	559

Furthermore, we identified a total of 125 microRNAs (miRNA), 261 small nuclear RNAs (snRNA), 718 transfer RNAs (tRNA), and 559 ribosomal RNAs (rRNA) within the 'Sanbai' genome (Table 1).

### Identification of SVs between 'Sanbai' and 'Tunisia'

**Figure 2:**
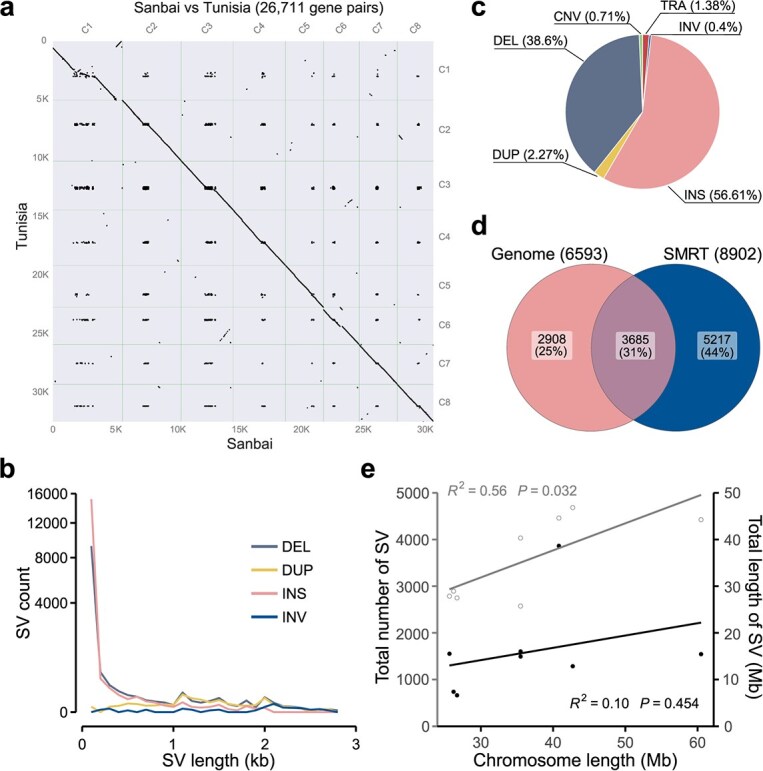
SVs identified by genome comparison between 'Sanbai' and 'Tunisia'. (a) Gene collinearity analysis between 'Sanbai' and 'Tunisia'. (b) The length distribution of different types of SVs is displayed on a square root scale, with the *y*-axis representing the number of SVs. (c) SVs detected by genome comparison between 'Sanbai' and 'Tunisia'. (d) A Venn diagram comparing the DELs, DUPs, and INVs detected by genome comparison and long-read mapping. (e) Correlation between chromosome length and the number of SVs (light gray) as well as SV lengths (dark gray)

By aligning the 'Tunisia' assembly to the 'Sanbai' genome, we discovered 26 711 homologous gene pairs within syntenic blocks between the two genomes (Fig. 2a; Table S4). Additionally, we identified 5892 genes unique to 'Sanbai' and 6651 genes exclusive to 'Tunisia' (Table S5). To detect SVs between 'Sanbai' and 'Tunisia', we employed two distinct methodologies (see section Materials and methods). The first approach involved a comparative analysis of the genome assemblies of 'Tunisia' and 'Sanbai', while the second method entailed aligning SMRT reads of 'Tunisia' to the 'Sanbai' genome assembly. The integration of both datasets unveiled a total of 28 607 non-redundant SVs, with an average sequence length of 4.45 kb and a maximum length of up to 5 Mb (Fig. 2b). Notably, approximately 5% (1441) of these SVs were large variants longer than 1 kb (Table S6), with chromosome 2 showcasing the highest number of SVs (Table S7).

The 28 607 identified SVs comprised 11 043 deletions (DELs), 650 duplications (DUPs), 16 197 insertions (INSs), 117 inversions (INVs), 205 copy number variations (CNVs), and 395 translocations (TRAs) (Table S6 and Fig. 2c). Of these, 6593 DELs, DUPs and INVs were detected by genome comparison, while 8902 DELs, DUPs and INVs were identified via SMRT read mapping (Fig. 2d). A comparison of the two methods revealed that 55.9% (3685) of the DELs, DUPs and INVs detected by genome comparison were also supported by SMRT read mapping, suggesting the credibility of our SV detection approaches. Furthermore, we observed a statistically significant correlation between the number of SVs on different chromosomes and their respective chromosome lengths (*R*^2^ = 0.562, *P* = 0.032) (Fig. 2e). However, no significant correlation was observed between SV length and chromosome length (*R*^2^ = 0.096, *P* = 0.45), aligning with previous findings in the Tibetan genome study [14].

### Population genetic analysis of 38 pomegranate accessions

**Figure 3:**
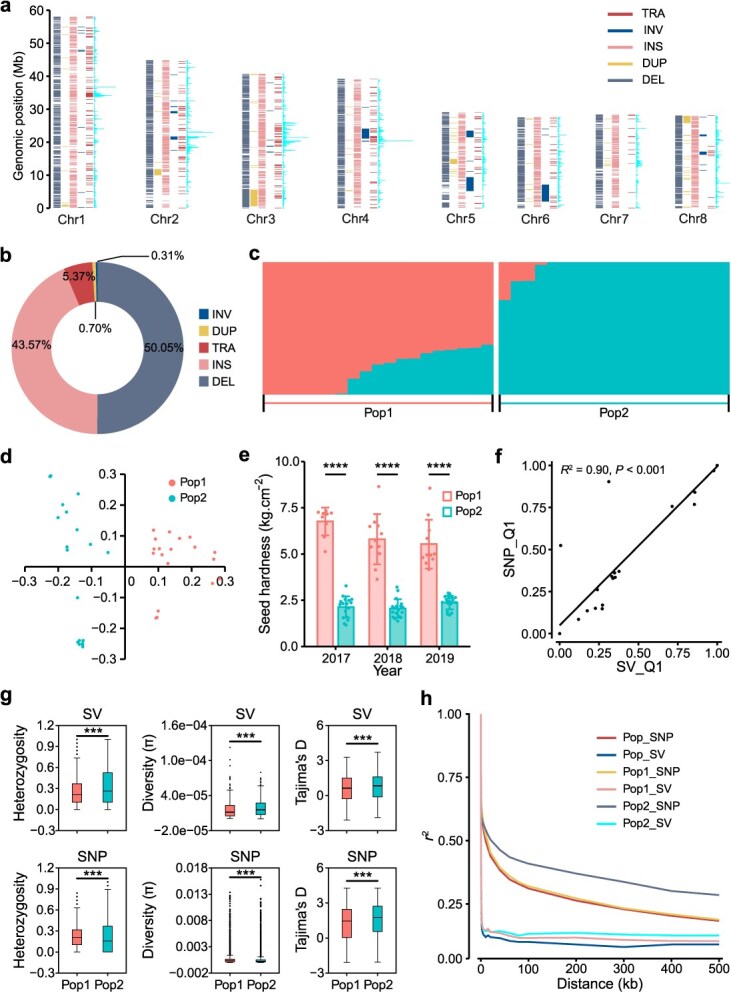
Population genetic analysis of 38 pomegranate accessions based on SVs and SNPs. (a) Chromosome distribution of translocations (TRAs), inversions (INVs), insertions (INSs), duplications (DUPs), and deletions (DELs) detected among the 38 pomegranate accessions. (b) The proportion of different types of SVs detected among the 38 pomegranate accessions. (c) Ancestry analysis (*K* = 2) of the 38 accessions based on SVs. (d) PCA of the 38 accessions based on SVs. (e) Comparison of seed hardness between Pop1 and Pop2 accessions. (f) Correlation analysis of the Q values for the 38 pomegranate accessions, calculated using SVs and SNPs. (g) Heterozygosity, nucleotide diversity (π), and Tajima's *D* values, calculated based on SVs (top panel) and SNPs (bottom panel), for Pop1 and Pop2. (h) The decay of LD, as measured by *r*^2^ based on SVs and SNPs, as a function of the physical distances between markers. The two colors in panels (c)–(e) represent groups Pop1 and Pop2, respectively, as indicated in panel (c)

The 28 607 SVs were used to genotype 38 pomegranate accessions using long-read resequencing data, resulting in the identification of 8085 polymorphic SVs (pSVs). Additional alignment of long-read resequencing data to the 'Sanbai' genome yielded 6154 pSVs. In total, we detected 14 239 pSVs, including 7127 DELs, 6204 INSs, 765 TRAs, 99 DUPs, and 44 INVs (Table S8; Fig. 3a and b). Admixture analysis based on these pSVs divided the 38 accessions into two distinct sub-populations, Pop1 and Pop2 (Fig. 3c), which was further supported by PCA and phylogenetic analyses (Fig. 3d and Fig. S7). Pop1 predominantly comprised hard-seeded and cold-tolerant accessions, while Pop2 mainly included soft-seeded accessions that were more susceptible to cold injury, as observed in our field trials (Fig. 3e). Concurrently, 428 815 single nucleotide polymorphisms (SNPs) were identified from short-read sequencing data of the 38 pomegranate accessions (Fig. S8). PCA and structure analyses based on SNPs were consistent with those obtained from pSVs (Fig. S9). The admixture proportions (*Q* values) derived from pSVs were significantly correlated with those from SNP data (*R*^2^ = 0.898, *P* = 2.2e-16) (Fig. 3f). These findings confirm the reliability of the detected SVs and highlight their roles in driving genetic divergence within pomegranates.

We next calculated nucleotide diversity (π), Tajima's *D*, and heterozygosity rate for Pop1 and Pop2 using both SNP and SV data. Both populations exhibited substantial genetic diversity, as reflected by nucleotide diversity (Fig. 3g). Tajima's *D* values exhibited uneven distribution across the genome, with mean values exceeding zero (Fig. 3g). The result implies either a historical decrease in population size or the influence of balancing selection on pomegranate, similar to patterns observed in other fruit trees like citrus [15]. Compared to Pop2, Pop1 accessions displayed a higher proportion of heterozygous variants based on SNP data (*P* < 0.01, Fig. 3g). This could be indicative of a more diverse gene pool in Pop1. In contrast, Pop2 may have undergone more intense selection pressures, which was further supported by the slower decay of linkage disequilibrium (LD) observed in Pop2 using either SNPs or SVs. Notably, the LD analysis based on SVs demonstrated a relatively faster LD decay over physical distances compared to SNP-based analysis (Fig. 3h), a pattern that has also been reported in Asian rice and grape studies [16, 17]. These genetic diversity parameters collectively highlight the distinct selective pressures experienced by Pop1 and Pop2 during domestication. In particular, the higher genetic diversity and faster LD decay observed in Pop1 suggest that this population may possess greater adaptability to changing environments, potentially aligning with enhanced cold tolerance.

### Contribution of SVs to a highly differentiated genomic region on chromosome 1 in pomegranate

**Figure 4:**
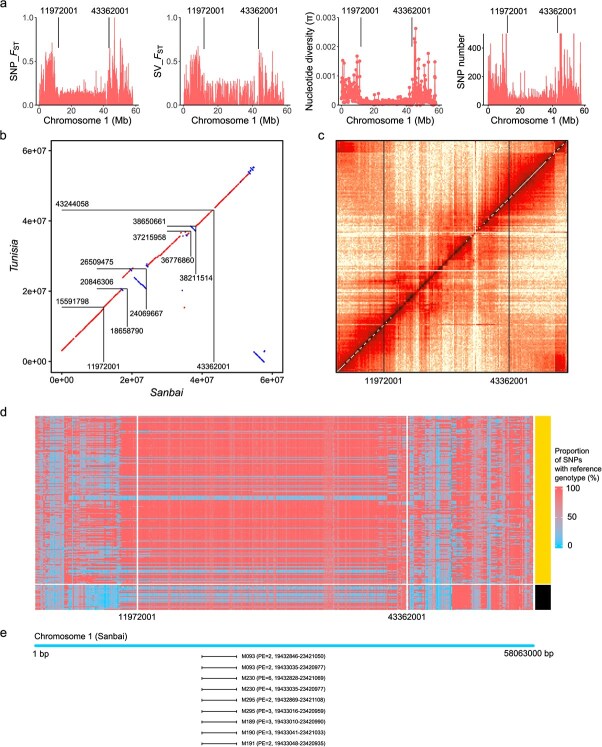
A highly differentiated genomic region on chromosome 1 of pomegranate. (a) *F*_ST_, nucleotide diversity (π) and SNP number in 100-kb non-overlapping genomic regions of chromosome 1. (b) Collinearity plot comparing chromosome 1 between 'Sanbai' and 'Tunisia'. (c) Hi-C interactions map for chromosome 1 of the 'Sanbai' genome. (d) Proportion of SNPs with the reference ('Sanbai') genotype in 100-kb genomic regions along chromosome 1 by aligning the short-read sequencing data of 286 pomegranate accessions to the 'Sanbai' genome. Each row represents a pomegranate accession, with hard-seeded and soft-seeded accessions distinguished by the color key on the right, respectively. (e) Visualization of paired-end reads from six representative accessions aligned to the 'Sanbai' genome, showing an ~5.4-Mb inversion (Chr 1: 18658790–24 069 667)

We previously identified an exceptionally large genomic region on chromosome 1 spanning approximately 26.2 Mb, which contributes to intragroup divergence, as delineated in the 'Tunisia' genome [6]. In this study, we further confirmed the presence of this large genomic region through selective sweep analysis using both SNP and SV data from 38 diverse pomegranate accessions (Fig. 4a). Our analysis revealed a considerable reduction in nucleotide diversity (π) and SNP count within this region compared to other segments on chromosome 1 (Fig. 4a). Comparative analysis of the genome assemblies of 'Sanbai' and 'Tunisia' led to the identification of two large inversions on chromosome 1 (Chr1: 18658790–24 069 667 and Chr1: 36776860–38 211 514), each exceeding 1 Mb in length (Fig. 4b), supported by the Hi-C interaction heatmap (Fig. 4c). Furthermore, re-sequencing data from 286 accessions aligned to the 'Sanbai' genome showed that within the large selective region, the proportion of SNPs exhibiting the 'Sanbai' reference genotype was consistently below 20% in the majority of cold-injury accessions. Conversely, for most cold-tolerant accessions, this proportion remained significantly above 80% (Fig. 4d). These findings provide plausible explanations for the markedly lower *F_ST_* and nucleotide diversity in this large genomic region, suggesting that selection has favored the 'Sanbai' genotype in cold-tolerant accessions.

To further explore the structural complexity of this region, we selected 12 representative accessions (three cold-tolerant accessions with a 'Sanbai'-like genotype, three cold-tolerant accessions with a 'Sanbai'-differing genotype, three cold-injury accessions with a 'Sanbai'-like genotype, and three cold-injury accessions with a 'Sanbai'-differing genotype) and aligned their Illumina short reads and SMRT long reads to the 'Sanbai' reference genome. This analysis revealed a ~5.4-Mb inversion (Chr1: 19432846–23 421 050) that largely overlaps with the previously identified large inversion (Chr1: 18658790–24 069 667). Notably, this inversion was present exclusively in accessions with genotypes significantly differing from 'Sanbai' in the large selective region (Fig. 4e), suggesting a potential role for this inversion in cold tolerance. Collectively, these results suggest that this large selective region on chromosome 1 is probably shaped by complex SVs, particularly inversions, which may impede recombination between cold-tolerant and cold-injury genotypes.

### Contributions of SVs to intra-group divergence in pomegranate

To identify potential genomic variations contributing to population differentiation during pomegranate domestication, we split the whole genome into 20-kb windows with a 2-kb step size and calculated the fixation index (*F*_ST_) between Pop1 (*n* = 19) and Pop2 (*n* = 19) for each window based on SVs and SNPs, respectively. By ranking the genomic windows based on their *F*_ST_ value and selecting the top 5% with the highest *F*_ST_ values, we identified 503 selective sweeps using SNPs and 336 selective sweeps using SVs, accounting for approximately 7.85% and 3.77% of the 'Sanbai' genome, respectively (Tables S9 and S10, Fig. S10a and b). A total of 3575 protein-coding genes were annotated within these selective sweeps (Table S11). Notably, approximately 69.7% (17.42 Mb) of SNP-based selection signals overlapped with SV-based selection signals (12 Mb) (Tables S9 and S10), encompassing 627 genes located in these overlapping selective sweeps. Among these, the MYB transcription factor (Pg02g59220) on chromosome 2 was identified within a selective sweep detected by both SNPs and SVs (Fig. S10a and b). In contrast, two AP2-like TFs (Pg03g141460, Pg04g177000), one NAC TF (Pg04g163740), and one WRKY TF (Pg04g173890) were detected exclusively in SV-based selective sweeps (Fig. S10a), highlighting the unique contribution of SVs to genomic variation. Additionally, eight genes were identified in SNP-based selective sweeps, including three *NAC* (Pg02g85170, Pg08g293430, Pg01g45590), two *MYB* (Pg01g46990, Pg05g201700), one *AP2*-like (Pg01g45790) and two forkhead box O (FoxO) signaling pathway genes (*Pg07g259140* and *Pg02g59410*) (Fig. S10b). The FoxO pathway is known to regulate genes involved in oxidative stress responses, apoptosis, and DNA repair, which are critical for plant survival under extreme environmental conditions [18, 19]. These findings suggest the importance of integrating SNP and SV data to capture the full spectrum of genetic diversity and provide a more comprehensive perspective on intragroup divergence in pomegranate.

### Identification of *PgNAC12* as a candidate gene for cold tolerance in pomegranate

**Figure 5:**
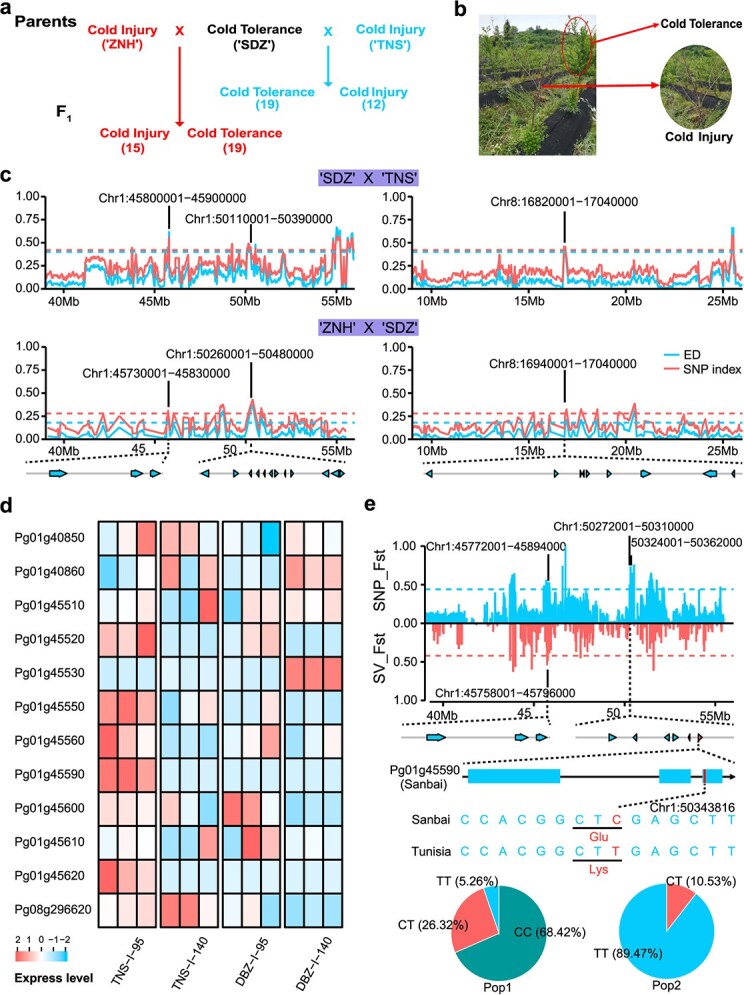
Identification of *PgNAC12* in the regulation of cold tolerance by bulk segregation analysis. (a) Design of two mapping populations for bulk segregation analysis, with the number of hybrid offspring used for bulk DNA sequencing indicated in parentheses. (b) Morphological comparison of two pomegranate varieties under cold stress conditions. (c) Bulk segregation analysis of the F_1_ population of 'SDZ' × 'TNS' and 'ZNH' × 'SDZ', utilizing Euclidean distance and SNP index metrics. (d) Expression levels (FPKM) of 12 candidate genes in the inner seed coats of 'Dabenzi' and 'Tunisia' at 95 or 140 days after flowering (DAF). The expression data were standardized using the scale function in R, by centering (subtracting the mean) and scaling (dividing by the standard deviation) for each gene. (e) Regional distribution of *F*_ST_ between Pop1 and Pop2 accessions across a genomic region on chromosome 1 (Chr 1: 39000000–56 000 000). Genes located in the candidate genomic region of Chr1: 45730001–45 900 000 and Chr1: 50272001–50 310 000 are indicated. The gene structure of Pg01g45590 and its haplotype analysis are displayed in the bottom panel

To unveil genes contributing to cold tolerance in pomegranate, we constructed two populations by crossing 'Shandazi' with 'Tunisia' ('SDZ' × 'TNS') and 'Zhongnonghong' with 'Shandazi' ('ZNH' × 'SDZ') (Fig. 5a and b), respectively. 'Tunisia' and 'Zhongnonghong' are cold-injury cultivars, while 'Shandazi' is a cultivar highly tolerant to cold stress. Utilizing bulk segregation analysis (BSA) of the F_1_ population of 'SDZ' × 'TNS', we successfully detected 41 genomic regions associated with cold tolerance (Table S12). Similarly, BSA analysis of the F_1_ population of 'ZNH' × 'SDZ' pinpointed 37 genomic regions related to cold tolerance, including two regions (Chr1: 21100001–21 340 000 and Chr1: 21600001–21 770 000) located within the large inversion on chromosome 1 (Table S13). Integration of the results from both populations revealed three candidate genomic regions (Chr1: 45730001–45 900 000, Chr1: 50110001–50 480 000, and Chr8: 16820001–17 040 000), designated as Pgcol1_1, Pgcol1_2, and Pgcol8, respectively (Fig. 5c). Within these regions, 24 protein-coding genes were annotated (Table S14). Of these, 12 genes were differentially expressed between the inner seed coats of hard-seeded 'Dabenzi' and soft-seeded 'Tunisia' at 95 or 140 days after flowering (DAF) (Fig. 5d; Table S14). These genes may be involved in both seed hardness and cold adaptation, though further confirmation of their roles is required.

**Figure 6:**
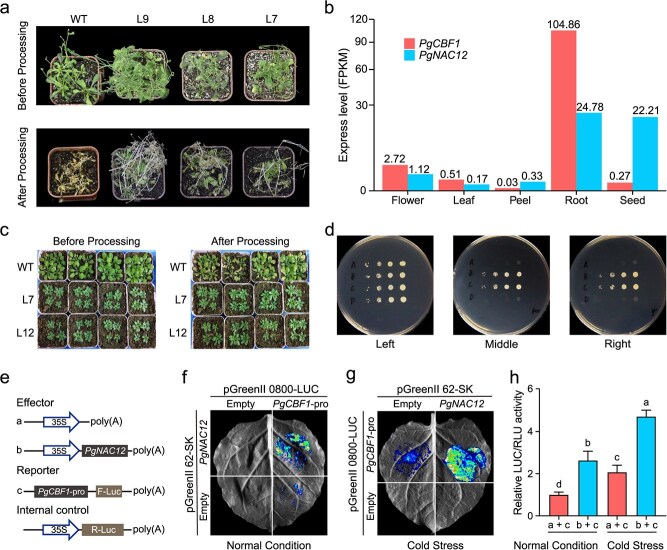
Functional validation of *PgNAC12* and *PgCBF1* in the regulation of cold tolerance. (a) Phenotypic comparison of WT and *PgNAC12* overexpression lines (L7, L8, and L9) in *Arabidopsis* under normal conditions (first panel) and cold stress conditions (second panel). (b) Expression levels of *PgCBF1* and *PgNAC12* (on a square root scale) in the flower, leaf, peel, root, and seed tissues of 'Sanbai'. (c) Phenotypic comparison of WT and *PgCBF1* overexpression lines (L7 and L12) in *Arabidopsis* under normal conditions (left panel) and cold stress conditions (right panel). (d) Co-transformation of the yeast strain Y1HGold and subsequent selection on selective media including SD/-Leu/-Ura medium (left panel), SD/-Leu/-Ura medium containing 300 ng/ml ABAr (middle panel) and SD/-Leu/-Ura medium containing 400 ng/ml ABAr (right panel). (e) Design of the construct for Luciferase transient transcriptional activity assay. (f-h) Luciferase activity assay showing that PgNAC12 interacts with the promoters of *PgCBF1*, with binding enhanced by cold stress. The binding ability of PgNAC12 to the *PgCBF1* promoter was indicated by the ratio of LUC to REN. Data are presented as mean ± SD of five biological replicates. Different letters indicate significant differences (*P* < 0.05) using Duncan's MRT after ANOVA

Selective sweep analysis unveiled that nine out of the 24 genes (*Pg01g40840, Pg01g40850, Pg01g40860, Pg01g45520, Pg01g45530, Pg01g45560, Pg01g45570, Pg01g45580,* and *Pg01g45590*) in Pgcol1_1 and Pgcol1_2 were located within selective sweeps detected in the present study (Fig. 5e). These genes may represent adaptive footprints in response to cold stress during domestication. Notably, Pg01g45590 was annotated as a NAC domain-containing transcription factor (PgNAC12), which showed high expression levels in the roots of ‘Sanbai’ (Fig. 5e). Further genetic analysis unveiled an SNP (Chr1: 50343816) in the exon of *PgNAC12* that distinguishes ‘Tunisia’ and ‘Sanbai’, resulting in the substitution of Glutamate (Glu) in ‘Sanbai’ with Lysine (Lys) in ‘Tunisia’. Importantly, this substitution is predominantly found in cold-sensitive accessions (89.47% of Pop2) (Fig. 5e). This observation underscores *PgNAC12* as a promising candidate related to cold tolerance in pomegranates.

### Functional validation of *PgNAC12* in regulating *PgCBF1* for cold tolerance

To further investigate the function of *PgNAC12*, we cloned the CDS sequence of *PgNAC12* and overexpressed it in *Arabidopsis* under the control of the CaMV35S promoter (Fig. S11a). Under cold stress, these overexpressing plants exhibited significantly greater tolerance compared to wild-type (WT) controls (Fig. 6a). Previous studies have shown that NAC TFs are well-documented for their crucial roles in cold stress responses through the CBF–COR signaling pathway in various plant species [20–22]. Expression analysis revealed that *PgCBF1* (*Pg01g43100*) was highly expressed in the roots of pomegranate, similar to PgNAC12 (Fig. 6b). Motivated by this insight, we further cloned the CDS sequence of *PgCBF1* and overexpressed it in *Arabidopsis* under the control of the CaMV35S promoter (Fig. S11b). As a result, the overexpressed lines exhibited robust growth, whereas the leaves of WT plants had turned yellow and withered (Fig. 6c). These results indicated that overexpression of *PgCBF1* significantly enhanced the cold resistance in *Arabidopsis*.

To delve deeper into the role of *PgNAC12* and *PgCBF1* in conferring cold stress tolerance, we verified the interaction between PgNAC12 and the promoter of *PgCBF1* through a yeast one-hybrid (Y1H) assay (Fig. 6d). The Y1H assay demonstrated that PgNAC12 directly binds to the *PgCBF1* promoter. To further quantify the binding capability of PgNAC12 to the *PgCBF1* promoter, we employed a dual-reporter plasmid system, which encompasses an LUC reporter driven by the *PgCBF1* promoter, an REN luciferase driven by the CaMV35S promoter, and an effector plasmid expressing *PgNAC12*, for transient dual-luciferase assays (Fig. 6e-g). The results indicate that PgNAC12 exhibits a significant activation effect on the *PgCBF1* promoter at room temperature, with the activation being further enhanced under cold conditions (Fig. 6f-g). These findings robustly confirm that *PgCBF1* is a direct target of PgNAC12, indicating that PgNAC12 can regulate the expression of *PgCBF1* through direct binding to its promoter. Moreover, the binding capability of PgNAC12 to the *PgCBF1* promoter is enhanced by cold exposure, suggesting a positive feedback loop that amplifies the cold response. Collectively, these findings highlight the critical roles of *PgNAC12* and *PgCBF1* in delineating the differentiation between soft-seeded and hard-seeded pomegranates in adaptation to cold stress (Fig. 6h). The differential expression of these genes could potentially explain the variation in cold tolerance observed between different pomegranate cultivars, providing valuable insights for breeding programs aimed at enhancing cold resistance.

## Discussion

### The high-quality genome assembly of 'Sanbai' marks a significant advancement in pomegranate genomics

Although two genome assemblies for hard-seeded pomegranate varieties ('Dabenzi' and 'Taishanhong') already exist, they remain fragmented due to their reliance on short-read sequencing technologies [2, 5]. These fragmented assemblies have proven insufficient for uncovering the full spectrum of genetic diversity, particularly in identifying structural variants and specific sequences associated with hard seed formation and cold tolerance. 'Sanbai', characterized by its hard seeds and freezing resistance, offers a valuable genetic resource for comparative genomics, enabling deeper insights into the genetic regulation of critical traits. In this study, we successfully assembled a high-quality, chromosome-level reference genome for 'Sanbai', which features an impressive contig N50 of 15.93 Mb and a scaffold N50 of 39.26 Mb (Table 1). Comprehensive evaluations, including BUSCO analysis, CEGMA analysis, and LAI estimates, confirmed the accuracy and completeness of the 'Sanbai' genome assembly (Fig. S3). This assembly fills a crucial gap in pomegranate genomics by providing a robust and reliable reference for hard-seeded and cold-resistant varieties. Notably, our study showcased the utility of this genome in identifying a ~5.4-Mb inversion on chromosome 1 (Fig. 4a and b). This inversion potentially plays a significant role in genetic differentiation related to cold tolerance among pomegranates by inhibiting recombination within this genomic region across different genotypes.

### Unveiling the role of SVs in pomegranate genomics and trait differentiation

SVs represent a relatively unexplored aspect of pomegranate genomics, despite their well-documented significance in shaping phenotypic diversity across various organisms [23–25]. Our study delves into the complex landscape of SVs in pomegranate, unveiling three crucial aspects that underscore their importance.

Firstly, we illuminate the prevalence of SVs within the pomegranate population employing both assembly-based and long-read mapping approaches (Fig. 2c and d). This represents the first comprehensive catalog of SVs for pomegranate, providing invaluable data for future studies on genetic diversity and trait association. Notably, the number of SVs identified in pomegranates is considerably lower compared to annual crops like maize [26, 27], rice [16], cotton [24], and pea [28]. This discrepancy may be attributed to the smaller genome size of pomegranates, which exhibits a correlation between chromosome length and SV occurrence (Fig. 2e). Additionally, the prevalence of self-pollination and clonal propagation in pomegranates could contribute to this trend [1].

Secondly, our findings highlight the influence of SVs on shaping pomegranate phenotype differentiation (Fig. 3c and d). Selection signals derived from SVs provide preliminary evidence of their involvement in cold adaptation, suggesting that SVs may play a crucial role in enabling certain pomegranate varieties to thrive in harsh environments. This has important implications for breeding programs aimed at enhancing cold tolerance in pomegranates, as SVs could be targeted to develop more resilient varieties.

Lastly, we conducted a comparative analysis of the effects of SVs versus SNPs on intra-specific pomegranate diversification. Our results indicate that SVs significantly contribute to population stratification within pomegranates (Fig. 3c, d and e), underscoring their importance in understanding population structure and differentiation. Moreover, SV datasets exhibited similar patterns of genome variability to SNPs, as evidenced by comparable linkage disequilibrium (LD) decay, nucleotide diversity, heterozygosity, and Tajima's *D* value among different sub-populations (Fig. 3g and h). Intriguingly, the combined analysis of SVs and SNPs uncovered a higher level of heritability compared to either variation type alone, demonstrating the complementary nature of these genetic variations.

### Genomic variations offer novel insights into the genetic mechanisms underlying cold tolerance in pomegranate

Low temperature is a critical environmental factor that significantly hinders the cultivation of soft-seeded pomegranates, with temperatures below seven degrees Celsius causing severe damage. Despite the importance of cold tolerance for the pomegranate industry, research into the genetic basis of this trait has been limited due to several challenges. Pomegranates are perennial woody fruit plants, which complicates the establishment of advanced genetic mapping populations, unlike annual crops such as rice or maize. Additionally, the underdeveloped state of genetic transformation techniques for pomegranates hinders functional studies of candidate genes. While our previous research has identified potential genetic factors under selection [6], refining the large set of candidate genes for further investigation has been challenging without additional data.

In this context, our study represents a significant advancement by employing a multi-population BSA approach to address these challenges. We identified three major QTLs and 24 candidate genes involved in cold stress response, with the *PgNAC12* transcription factor emerging as a particularly significant finding. Through molecular biology techniques and genetic transformation, we demonstrated that *PgNAC12* positively regulates *PgCBF1*, thereby enhancing cold tolerance in pomegranates. These findings not only deepen our understanding of the genetic mechanisms underlying cold resistance but also open new avenues for targeted genetic improvement in soft-seeded pomegranates.

## Conclusion

In this study, we present a significant advancement in pomegranate genomics by delivering a high-quality, chromosome-level genome assembly for the 'Sanbai' pomegranate variety. This assembly fills a critical gap in pomegranate genomics, providing a robust reference for future comparative and population genomics studies. The 'Sanbai' genome has facilitated the identification of SVs, offering a comprehensive view of genetic diversity, population differentiation, and breeding processes in pomegranates. Furthermore, our innovative multi-population BSA approach has enabled us to pinpoint QTLs associated with cold tolerance. Through genetic transformation experiments, we have demonstrated that *PgNAC12* positively regulates the expression of *PgCBF1*, thereby enhancing cold tolerance in pomegranates. These findings offer valuable insights into the genetic mechanisms underlying cold tolerance, particularly in soft-seeded varieties.

## Materials and methods

### Sample preparation and sequencing

'Sanbai' is a Chinese pomegranate landrace collected from Shandong Province of China. Other pomegranate accessions used in this study were cultivated in a nursery at the Zhengzhou Fruit Research Institute, Chinese Academy of Agricultural Sciences, Henan province, China. Young leaves from each cultivar were collected and immediately frozen in liquid nitrogen. Genomic DNA was then extracted from these leaves, and its quality was verified through pulsed-field gel electrophoresis.

DNA from 'Sanbai' was used to prepare sequencing libraries following the methods described by Chin *et al.* [29], which were sequenced on the PacBio (Pacific Biosciences) Sequel platform using Sequel Sequencing chemistry v.3.0. Sequencing with one SMRT cell yielded 56.13 Gb of raw data. After filtering, 2 752 901 clean PacBio sequencing reads with an N50 of 20.37 kb were obtained.

For ultra-long read sequencing, genomic libraries were prepared using the Oxford Nanopore Sequencing kit and sequenced using the Oxford Nanopore MinION (ONT) platform. For Illumina sequencing, paired-end libraries were prepared following the manufacturer’s protocol (Illumina, San Diego, CA) and sequenced using the Illumina HiSeq 2500 platform.

For Hi-C sequencing, a mixture of root, fruit peel, seed, flower and leaves of 'Sanbai' were used for DNA extraction and library preparation.

### Whole genome sequencing of pomegranate accessions

For each of the 286 pomegranate accessions collected in this study, genomic DNA was extracted from young leaves and whole-genome resequencing was performed using the Illumina HiSeq 2500 platform. From these 286 pomegranate accessions, 38 representative accessions were selected for genome re-sequencing utilizing the Oxford Nanopore platform.

### *De novo* genome assembly and evaluation

Genome assembly was initially performed using NextDenovo (v2.5, https://github.com/Nextomics/NextDenovo), with parameters “--genome_size 30m --read_type clr” for SMRT reads. Then, the racon package was employed to correct the assembly using the PacBio raw reads with default parameters. The assembly was further polished for four times by Nextpolish (default parameters, https://github.com/Nextomics/NextPolish.git) using Illumina short reads. The resulting contigs were then used to build scaffolds by ALLHiC (v0.9.8, https://github.com/tangerzhang/ALLHiC) using Hi-C sequencing data.

Genome completeness was evaluated using the embryophyta BUSCO dataset (v.3.0) and the CEGMA database (v2.5) [30]. Illumina short-reads were mapped to the assembled genome using BWA (v0.7.8) [31] to assess coverage and read depth. The LTR Assembly Index (LAI) program within the LTR_retriever package (v1.0.7) was used to evaluate genome continuity [13]. The collinear relationship between 'Sanbai' and 'Tunisia' was visualized using dot plots generated by nucmer and mumplot from MUMmer v.3.23 [32] with the parameters: -l 100 -c 1000 -d 10 -banded -D 5.

### Annotation of transposons and protein-coding genes

We employed RepeatMasker (v4.10) to annotate repetitive elements in the 'Sanbai' genome sequence. Tandem repeats were identified using Tandem Repeat Finder (v.4.09) [33]. For protein-coding gene annotation, we employed a multifaceted approach. Initially, we conducted ab initio gene predictions using Augustus (v3.2.3) [34] and SNAP (v.2006.07.28.) [35]. For homolog-based predictions, we used TBLASTN (v2.2.26) with an E-value threshold of <10e-5. In addition, for transcript-based prediction, RNA-seq data were mapped to the reference genome using Hisat2 (v2.0.4), and transcripts were assembled using Stringtie (v1.2.3). GeneMarkS-T (v5.1) was then employed to predict genes based on the assembled transcripts. To further refine gene predictions, we utilized PASA (v2.2.0) [36] to incorporate unigenes and full-length transcripts obtained from PacBio sequencing, which were assembled using Trinity (v2.1.1). All gene models generated from these diverse approaches were integrated using EVidenceModeler (EVM, v1.1.1, http://evidencemodeler.github.io/). Additionally, we predicted microRNAs, rRNAs, and tRNAs using the miRBase, Rfam, and tRNAscan-SE (version 1.3.1) databases [37–39]. Small nuclear RNAs (snRNAs) were predicted using INFERNAL against the Rfam database.

### Identification of synteny genes

To identify synteny blocks, we first conducted genome alignment using the MUMmer program (v.3.2) with the following command: nucmer --mum --maxgap=500 --mincluster=1000. Simultaneously, we conducted an all-by-all BLASTP (v.2.2.26) comparison of protein sequences to identify homologous genes, using an E-value cutoff of ≤1e−7 and an identity threshold of ≥20%. Subsequently, we employed BEDTools (v.2.27) to find intersections between these sets of homologous genes, specifically focusing on those within one-to-one genomic synteny blocks. Genes identified as homologous within these synteny blocks were classified as syntenic genes.

### Detection of genetic variations

Structural variations were detected employing both assembly-based and read-mapping-based approaches. To identify SVs between the 'Sanbai' and 'Tunisia' primary assemblies, we conducted an assembly comparison using minimap2 and SyRI [40]. Furthermore, we aligned PacBio long-read data from 'Tunisia' to the 'Sanbai' genome to identify additional SVs. This was accomplished using Sniffles and NGMLR (v.0.2.7) for SV detection, based on long reads alignments generated by Minimap2. The NucDiff tool [41] was then employed to extract features and coordinates of the SVs. Subsequently, we compared the genomic locations of these SVs using bedtools (v.2.25) and combined SVs with a minimum reciprocal overlap of 80%. Here, we focused on identifying SVs greater than 10 bp, in accordance with similar researches conducted in maize [22]. The genomic distribution of SVs and other genome elements was visualized using a Circos plot created with shinyCircos-V2.0 [42].

For SNP detection, we aligned Illumina whole-genome resequencing data to the 'Sanbai' reference assembly. SNP calling was performed using the HaplotypeCaller tool within the Genome Analysis Toolkit (GATK v.4.0) pipeline. To ensure high data quality, we applied SNP filtering using the VariantFiltration module with the filter criteria: “QD < 2.0 || MQ < 40.0 || FS > 60.0 || SOR > 3.0 || MQRankSum < -12.5 || ReadPosRankSum < -8.0 || ExcessHet > 54.69”. Additionally, we required that each SNP site have no more than 20% missing values and at least 5% minor allele frequency.

### Population genetic analysis

We employed a sliding window of 10 kb with a step size of 5 kb to estimate Tajima's *D* values for each sub-population (Pop1 and Pop2) along the genome. Nucleotide diversity (π) for Pop1 and Pop2, as well as the fixation index (*F*_ST_) between populations, were computed using VCFtools (v0.1.14) [43] within a 100 kb window. A neighbor-joining (NJ) tree was constructed using the TreeBest software (v1.9.2) [44] with 1000 bootstrap replications. Principal component analysis (PCA) was performed using the GCTA software [45]. Linkage disequilibrium (LD) was calculated using PLINK (version 1.07, [46]) with the parameter '--file --r2 --ld-window 99999 --ld-window-kb 200 --out'. The ADMIXTURE program (v1.23) was used to estimate the ancestry proportions of each pomegranate accession [47].

### Selective sweep analysis

To identify evidence of selection in pomegranate genomes, we calculated the Fixation Index (*F*_ST_) between Pop1 and Pop2 using VCFtools (v0.1.14) within 20-kb sliding windows and a 2-kb step size [43]. Candidate selected regions were identified by selecting the top 5% of *F*_ST_ scores within these windows.

### BSA analysis

We conducted BSA analysis using two methods: SNP-index and Euclidean distance. The SNP-index was calculated as the ratio of reads corresponding to a mutant SNP to the total number of reads covering that SNP. This index equals 1 when all short reads differ from the reference genome and 0 when all short reads match the reference genome. For projects involving two offspring pools, SNPs with SNP-indices less than 0.3 in both pools were filtered out. After filtering, we calculated the average SNP-index within a 100-kb sliding window with a 10-kb step size. The difference in SNP-index between the two pools was denoted as △SNP-index, and the absolute value of △SNP-index was used as suggested by Xue *et al.* [48]. The Euclidean distance (ED) method was used to identify loci with significant differences between mixed pools and to evaluate regions associated with specific traits. SNP sites with genotype differences between the two offspring pools were used to calculate the depth of each base in different mixed pools., and the ED value for each site was calculated. In this project, we squared the original ED values to enhance the associated signal and minimize background noise. The average ED value was then calculated within a 100-kb sliding window with a 10-kb step size.

For both methods, windows in the top 5% of scores were considered as candidate regions. Common regions identified by both the SNP-index and ED methods were designated as candidate quantitative trait locus (QTL) regions.

### Yeast one-hybrid assay

The *PgCBF1* promoter (a 1800-bp fragment upstream of the start codon) was cloned into the SacI/SalI-digested pAbAi vector to generate the pAbAi-pPgCBF1 construct. The CDS of PgNAC12 was cloned into the EcoRI/BamHI-digested pGADT7 vector to obtain pGADT7-PgNAC12 construct. The Y1H assay was performed according to the manual of Matchmaker Gold Yeast One-Hybrid Library Screening System (Clontech). The pAbAi-pPgCBF1 construct served as the bait and was transformed into the Y1HGold yeast strain. The p53 construct was used as a positive control. The pGADT7-PgNAC12 plasmid was introduced into the bait strains. Co-transformed yeast cells were cultured on SD/−Leu-Ura agar plates, with or without ABA, and incubated at 30°C for 3–5 days.

### Construction of overexpression vector and genetic transformation of *Arabidopsis*

The CDS of the candidate genes was cloned into the pBI121 vector at XbaIsites to generate the 35S-candidate gene construct. The recombinant *Agrobacterium tumefaciens GV3101* strain harboring the 35S-candidate gene construct was then transformed into *Arabidopsis* [49]. Seeds were germinated on agar plates containing half-strength Murashige and Skoog’s medium (1/2 MS) with 0.7% (w/v) agar, 1% (w/v) sucrose at pH 5.8 and kanamycin (50 mg/L). A cold-tolerance assay was performed at −10°C for 4 hours using WT and homozygous overexpression lines selected from transgenic plants. After a 10-day recovery period under normal conditions, the phenotypic differences between the WT and transgenic plants were compared.

### Dual-luciferase assay

To analyze the binding capacity of PgNAC12 to the *PgCBF1* promoter, specific primers were employed to amplify the *PgCBF1* promoter region and the PgNAC12 CDS via PCR. These fragments were subsequently cloned into the dual-reporter vectors pGreenII 0800-LUC and pGreenII 62-SK, respectively. The recombinant vectors were transformed into the *A. tumefaciens* strain GV3101 (pSoup19). The transformed *A. tumefaciens* cells were resuspended in MES and MgCl2 solution and co-injected into tobacco leaves. The binding ability of PgNAC12 to the *PgCBF1* promoter under cold stress and room temperature conditions was quantitatively analyzed using a chemiluminescence detection system (Promega).

## Supplementary Material

Web_Material_uhaf022

## Data Availability

All datasets have been deposited in National Genomics Data Center (NGDC) with the following accession codes: genome assembly and gene annotation of 'Sanbai', GWHEQOV00000000; genome resequencing of 38 pomegranate accessions using the Illumina platform, CRA021734; genome resequencing of 38 pomegranate accessions using the Oxford Nanopore platform, CRA021775; and genome resequencing data for Bulked Segregant Analysis, CRA021764.
