# Supplementary material for: Genome assembly of pomegranate highlights structural variations driving population differentiation and key loci underpinning cold adaption
Source: Hortic Res. 2025 Jan 21;12(5):uhaf022. doi: 10.1093/hr/uhaf022 (PMC11979328; doi:10.1093/hr/uhaf022)
Supplement: Web_Material_uhaf022 [file web_material_uhaf022.zip › Supplementary File - Figures.docx]

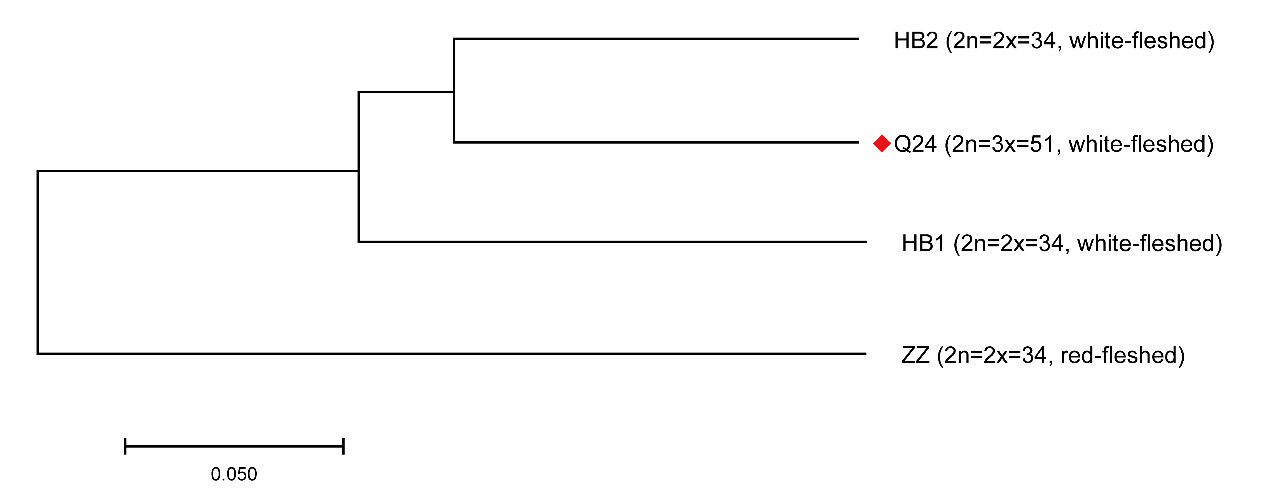


**Fig. S1** **Phylogenetic tree of 4 parents constructed by neighbor joining method based on genetic distances.** Genetic distances were calculated on SNPs information from whole genome re-sequencing data. Q24 and HB2 were derived from ‘Changbai 1’ as female parent, so genetic distance of them was 0.179. HB1 was derived from ‘Ruantiaobaisha’, and the genetic distance between Q24 and HB1 was 0.227. ZZ was a hybrid of a China native loquat variety ‘Jiefangzhong’ and a Japan variety ‘Moriowase’, and the genetic distance between Q24 and ZZ was 0.382. HB1 indicates ‘Huabai 1’; HB2 indicates ‘Huabai 2’; ZZ indicates ‘Zaozhong 6’.


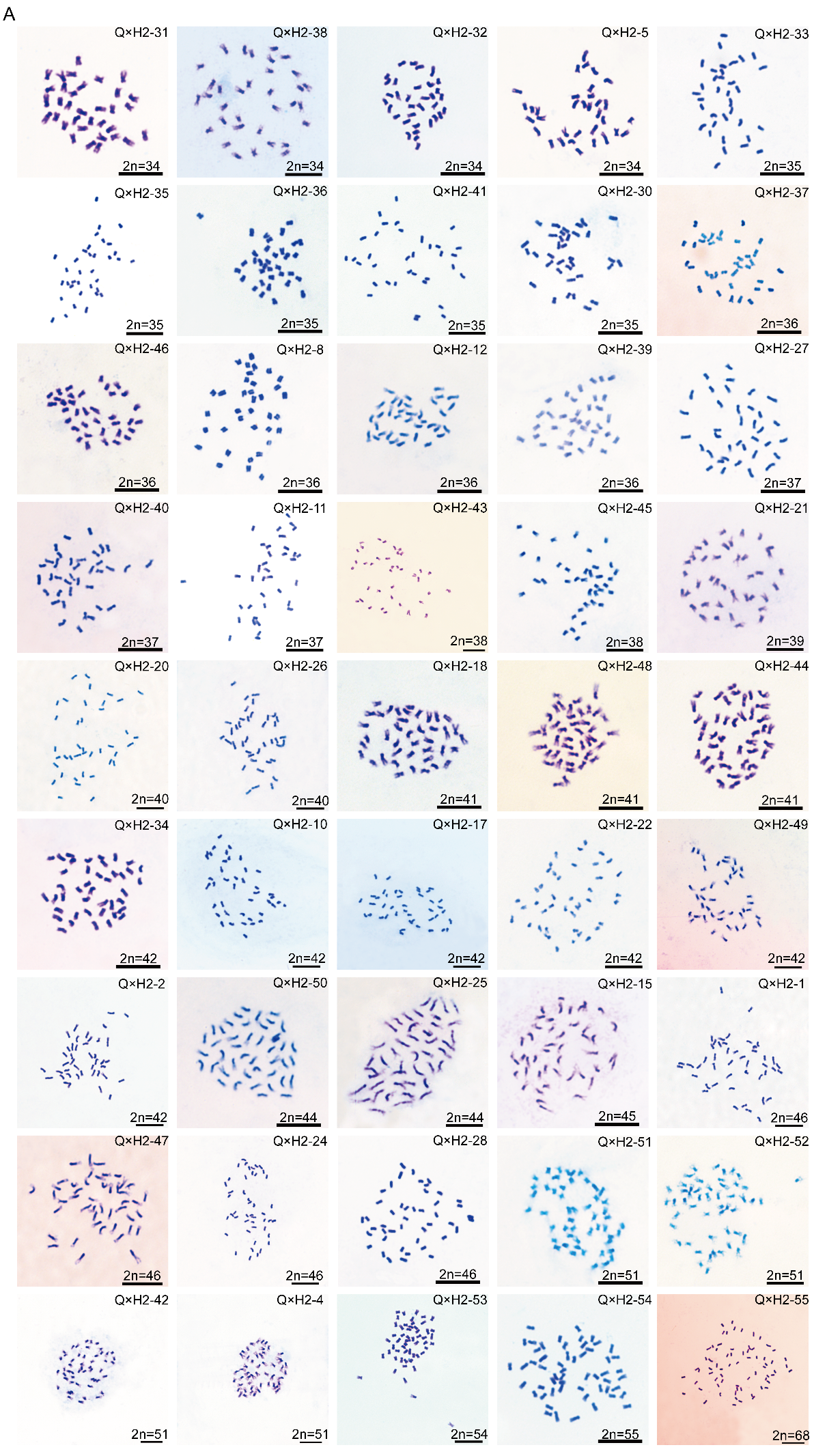


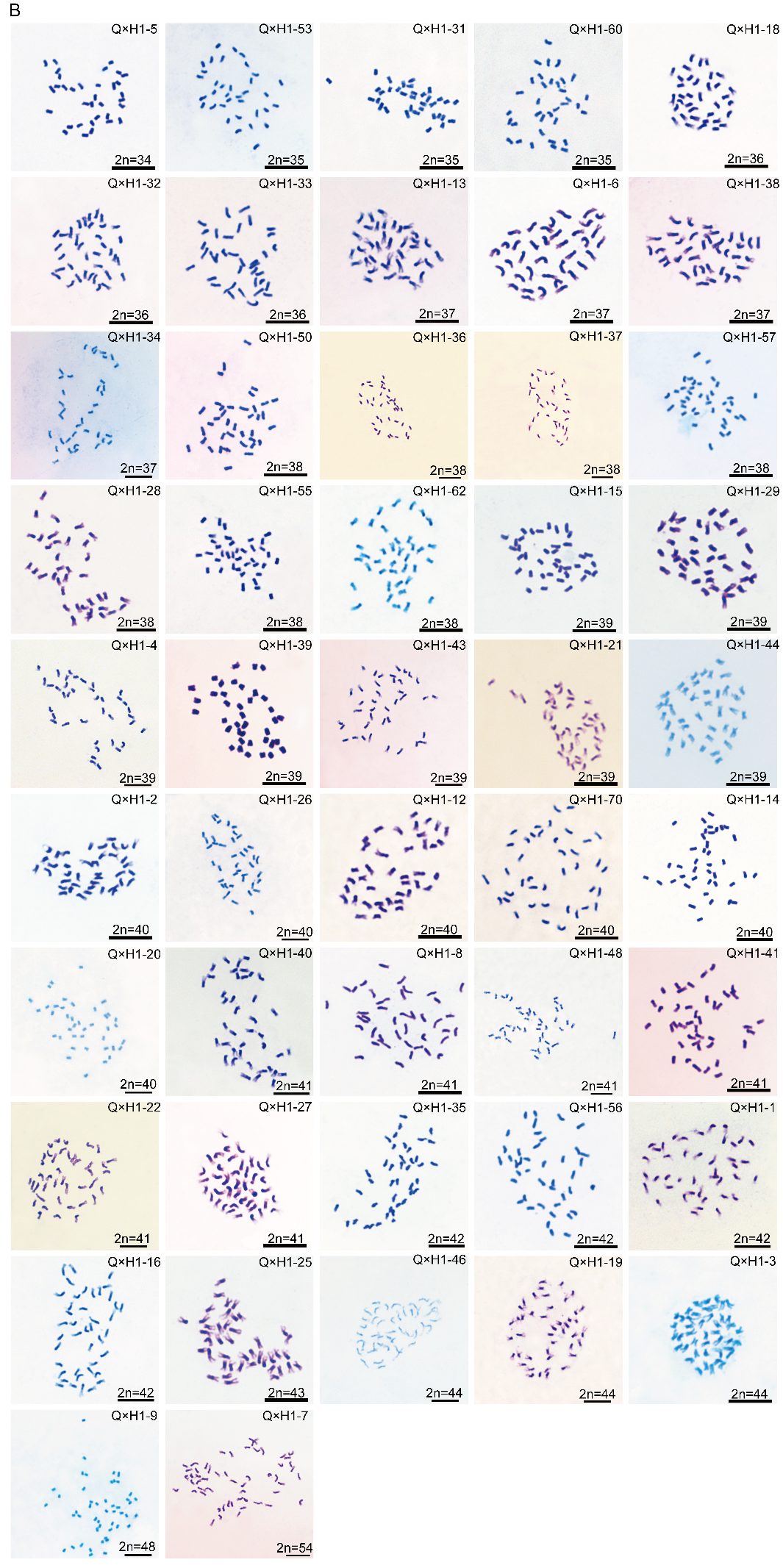

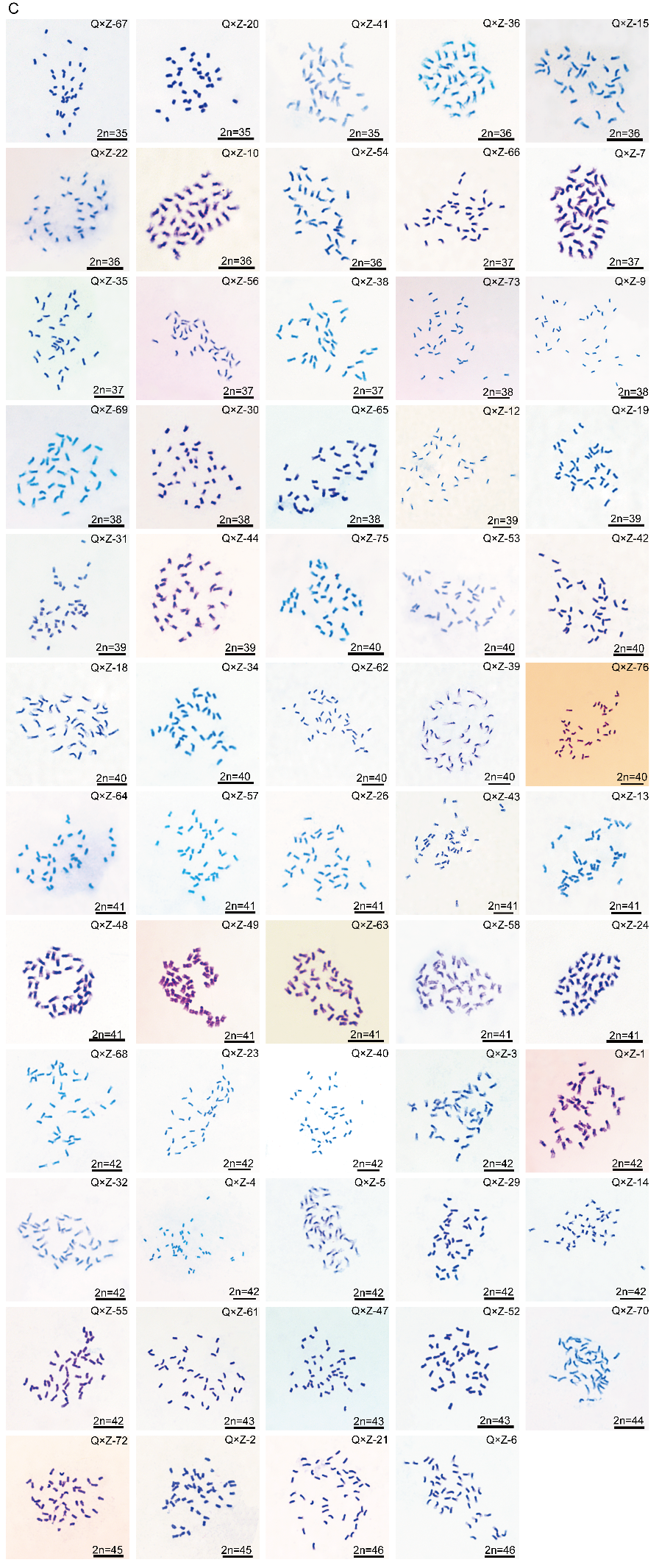


**Fig. S2** **Chromosome photographs of the hybrid progenies of triploid loquat Q24. A** The chromosomes of the F1 progenies derived from Q24 × Huabai 2 cross combination. **B** The chromosomes of F1 progenies derived from Q24 × Huabai 1 cross combination. **C** The chromosomes of the F1 progenies derived from Q24 × Zaozhong 6 cross combination. The labels of plants were in the upper right corners of the chromosome photographs, and the chromosome numbers are showed in the lower right corner above of the bars. Bars=10 μm.


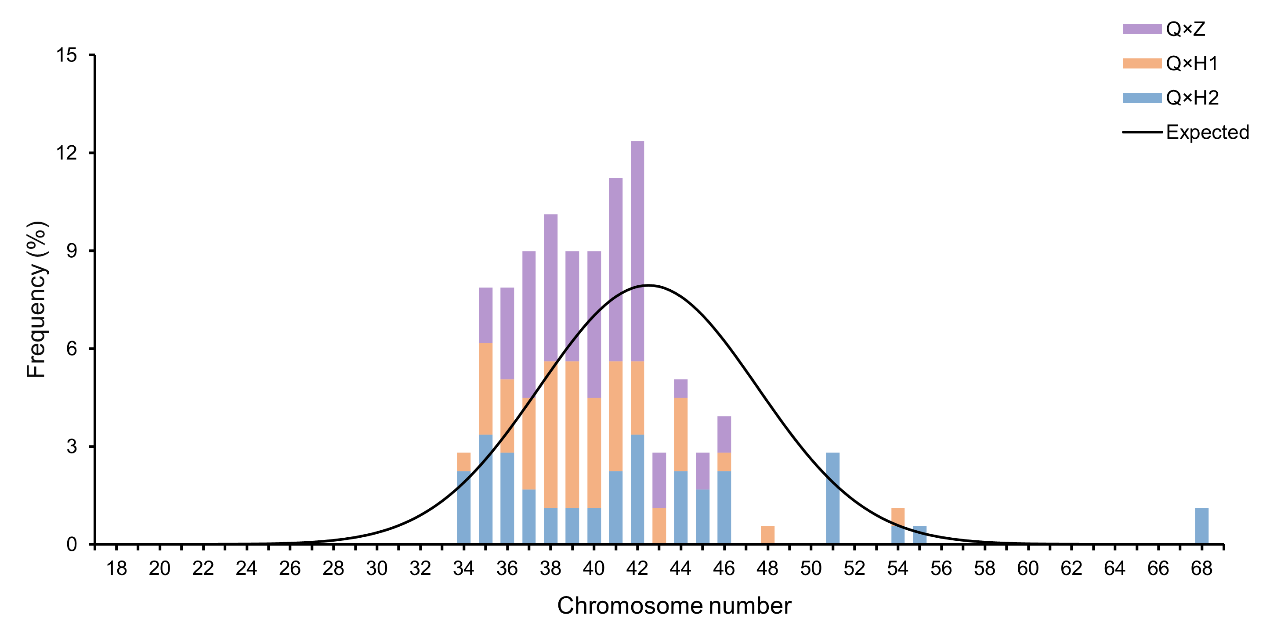


**Fig. S3 The distribution of chromosome number of hybrid plants in three hybrid combinations.** The column diagram indicates the actual distribution of chromosome numbers. The curved line indicates the expected distribution of the chromosome numbers (normal distribution based on random chromosome segregation) in the offspring of the triploid loquat and diploid loquat hybrids, with 42.5 as the mean value and a variance equal to the actual distribution. The variances were 5.03, respectively.


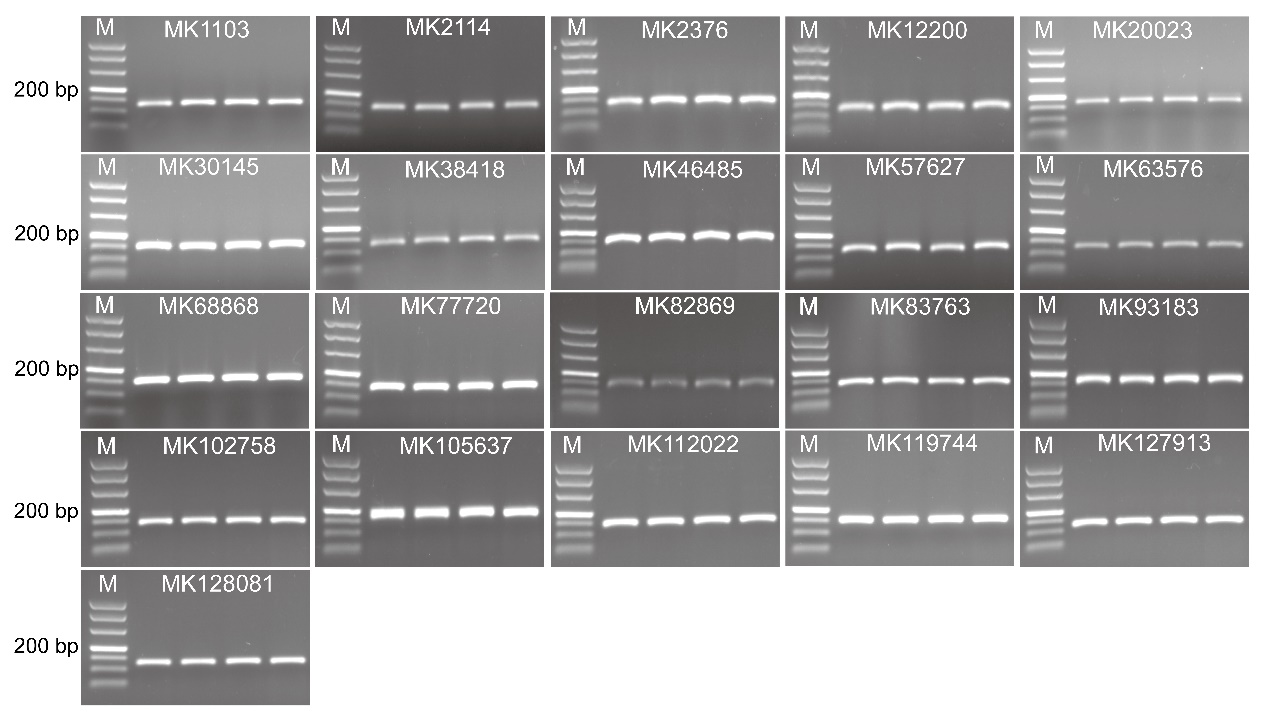


**Fig. S4 Agarose gel electrophoretogram of products amplified from 4 parents genomic DNA by 21 specific SSR primers.** The primer ID are noted above the gel photos. M represents DL 500 DNA marker. The lanes from left to right in every photo are respectively Q24, ‘Huabai 1’, ‘Huabai 2’, and ‘Zaozhong 6’.


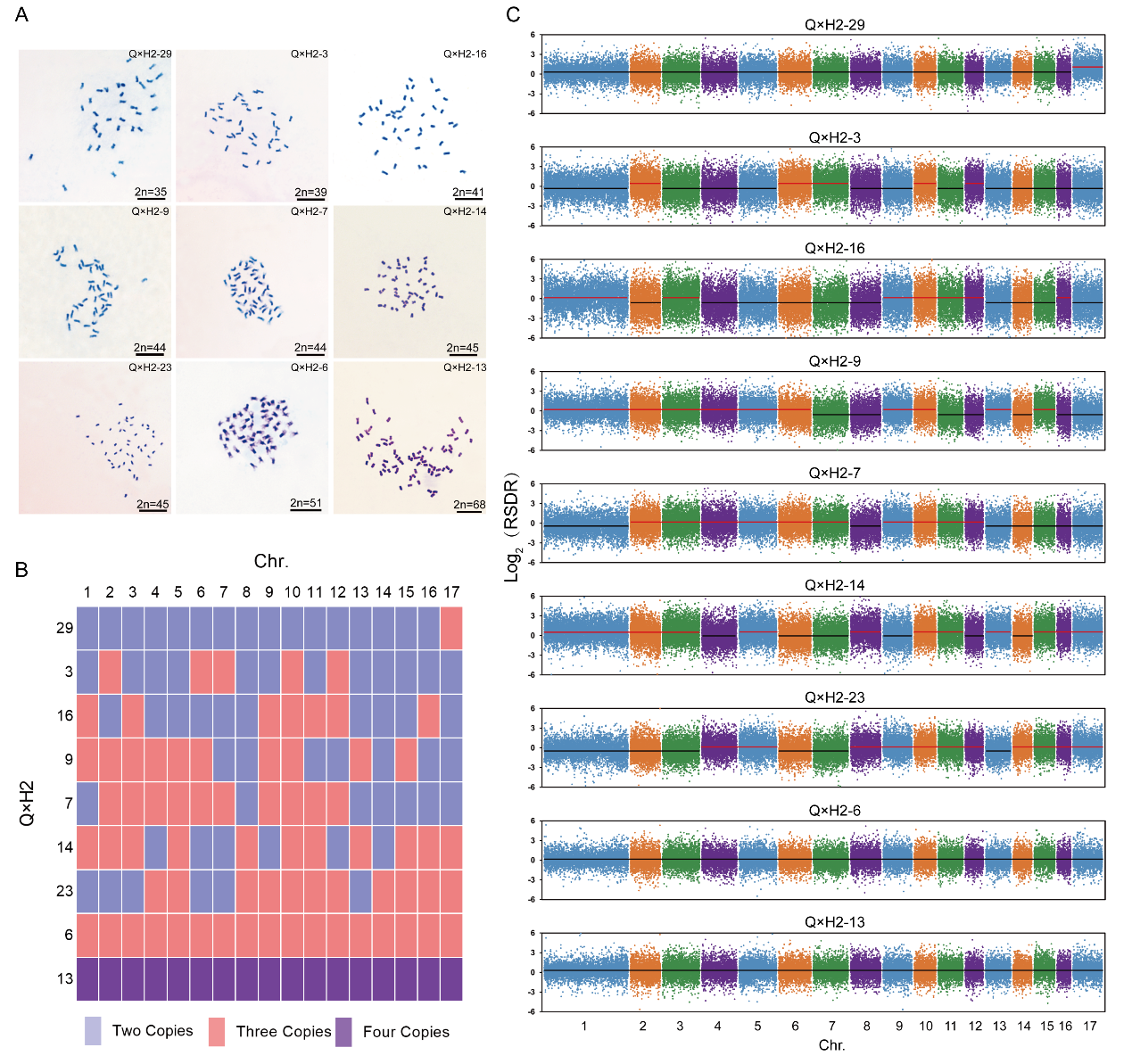


**Fig. S5 Verification of the improvement SSR-qPCR method for identifying aneuploid loquat karyotypes of Q×H2 progenies. A** Mitotic metaphase chromosome of nine aneuploids from Q×H2 cross. The labels of plants were in the upper right corner of the chromosome photograph, and the chromosome numbers are showed in the lower right corner above of the bars. Bars=10 μm. **B** Molecular karyotype of the nine aneuploid loquat strains identified by the improved SSR-qPCR method. The molecular karyotype pattern of aneuploid loquat was constructed. The plant labels are showed on the left of the block diagram. Every block indicates one chromosome, and chromosome numbers are noted on the upper side. The blue blocks indicates that there were two chromosome copy numbers. The red block shows three chromosome copy numbers. And purple block showed that there are four chromosome copy numbers. The chromosome numbers of plants were counted as: 2 × numbers of blue block + 3 × numbers of red blocks+ 4 × numbers of purple blocks. **C** The ddRAD-seq was used for molecular karyotype identification of aneuploid loquats. Each dot shows the log_2_(RSDR) value of every SNP locus on chromosomes. SNP were sorted according to their position on the chromosome, at equidistance. The plant labels are noted on the top of every scatter diagram, and the chromosome No. appear on the bottom of these scatter diagrams. The black and red horizontal solid lines represent normal chromosomes and abnormal chromosomes.


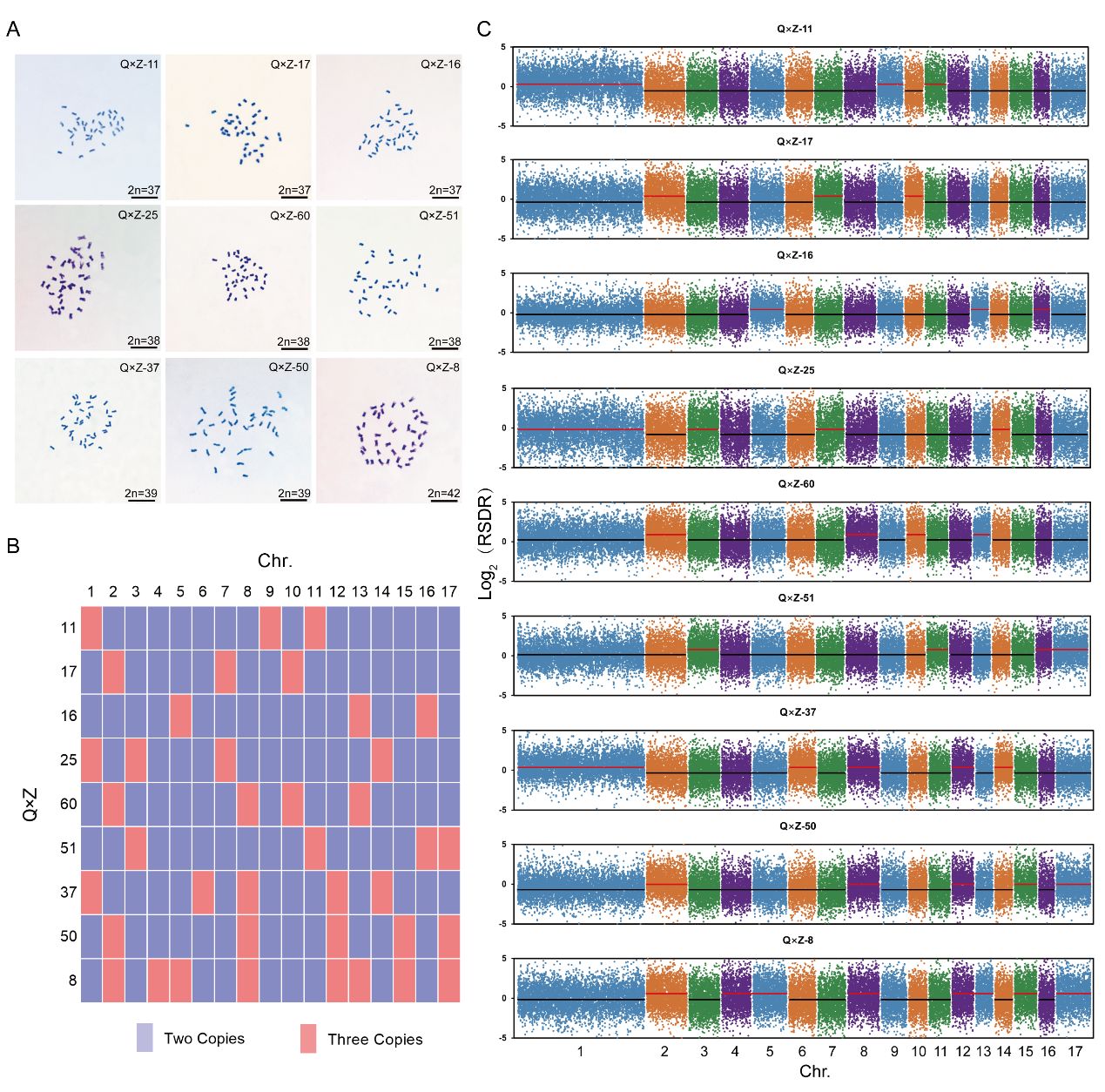


**Fig. S6 Verification of the improvement SSR-qPCR method for identifying aneuploid loquat karyotypes of Q×Z progenies. A** Mitotic metaphase chromosomes of nine aneuploids from Q×Z cross. The labels of plants were in the upper right corner of the chromosome photograph, and the chromosome numbers are showed in the lower right corner above of the bars. Bars=10 μm. **B** Molecular karyotype of the nine aneuploid loquat strains identified by the improved SSR-qPCR method. The molecular karyotype pattern of aneuploid loquat was constructed. The plant labels are showed on the left of the block diagram. Every block indicates one chromosome, and chromosome numbers are noted on the upper side. The blue blocks indicates that there were two chromosome copy numbers. The red block shows three chromosome copy numbers. The chromosome numbers of plants were counted as: 2 × numbers of blue block + 3 × numbers of red blocks. **C** The ddRAD-seq was used for molecular karyotype identification of aneuploid loquats. Each dot shows the log_2_(RSDR) value of every SNP locus on chromosomes. SNP were sorted according to their position on the chromosome, at equidistance. The plant labels are noted on the top of every scatter diagram, and the chromosome No. appear on the bottom of these scatter diagrams. The black and red horizontal solid lines represent normal chromosomes and abnormal chromosomes.


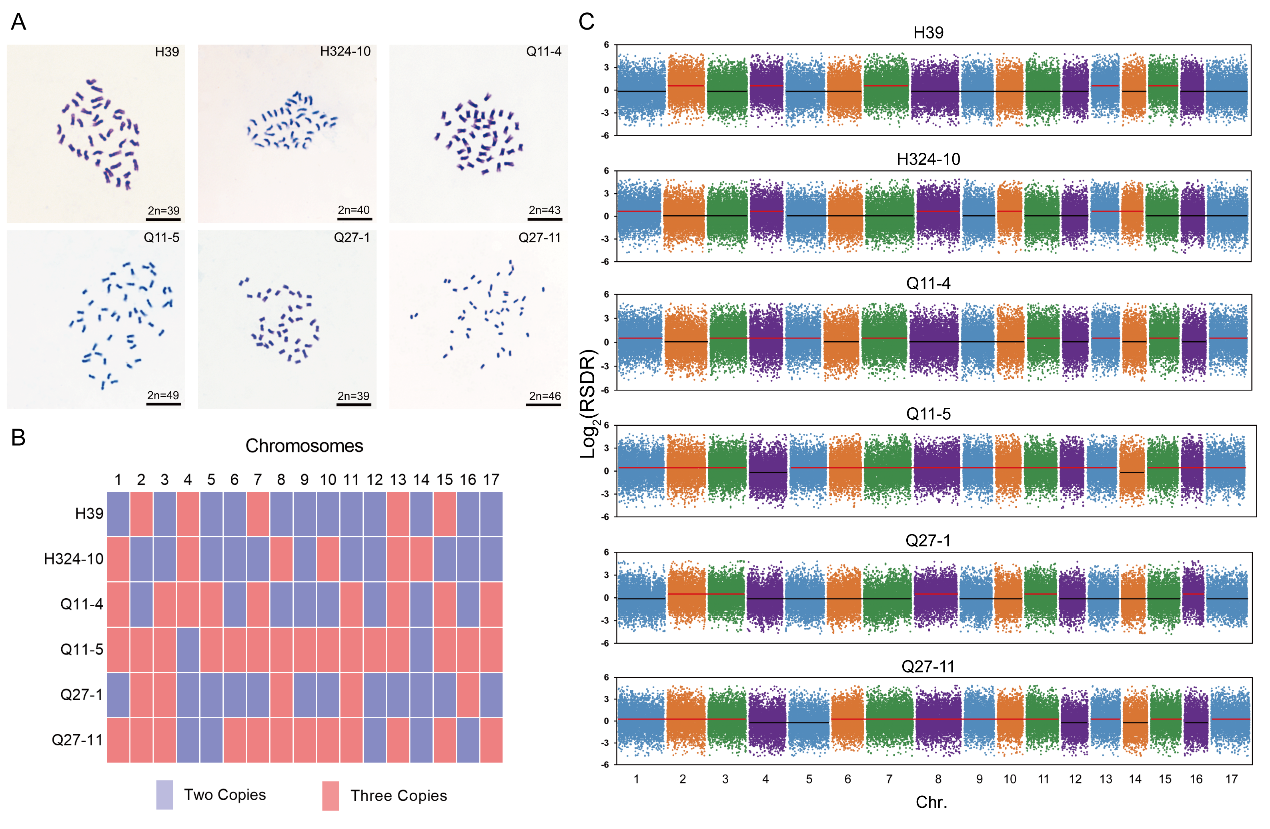


**Fig. S7 Verification of the improvement SSR-qPCR method for identifying aneuploid loquat karyotypes in different types of triploid loquat offspring. A** Mitotic metaphase chromosomes of six aneuploids from different types of triploid loquat offspring. The labels of plants were in the upper right corner of the chromosome photograph, and the chromosome numbers are showed in the lower right corner above of the bars. Bars=10 μm. **B** Molecular karyotype of the six aneuploid loquat strains identified by the improved SSR-qPCR method. The molecular karyotype pattern of aneuploid loquat was constructed. The plant labels are showed on the left of the block diagram. Every block indicates one chromosome, and chromosome numbers are noted on the upper side. The blue blocks indicates that there were two chromosome copy numbers. The red block shows three chromosome copy numbers. The chromosome numbers of plants were counted as: 2 × numbers of blue block + 3 × numbers of red blocks. **C** The ddRAD-seq was used for molecular karyotype identification of aneuploid loquats. Each dot shows the log_2_(RSDR) value of every SNP locus on chromosomes. SNP were sorted according to their position on the chromosome, at equidistance. The plant labels are noted on the top of every scatter diagram, and the chromosome No. appear on the bottom of these scatter diagrams. The black and red horizontal solid lines represent normal chromosomes and abnormal chromosomes. Note, H39 and H324-10 was derived from the offspring of triploid ‘Wuheguoyu’. Q11-4 and Q11-5 was descendants of triploid ‘Wuhezaoyu’. Q27-1 and Q27-11 was derived from the offspring of triploid ‘Huayuwuhe 1’.


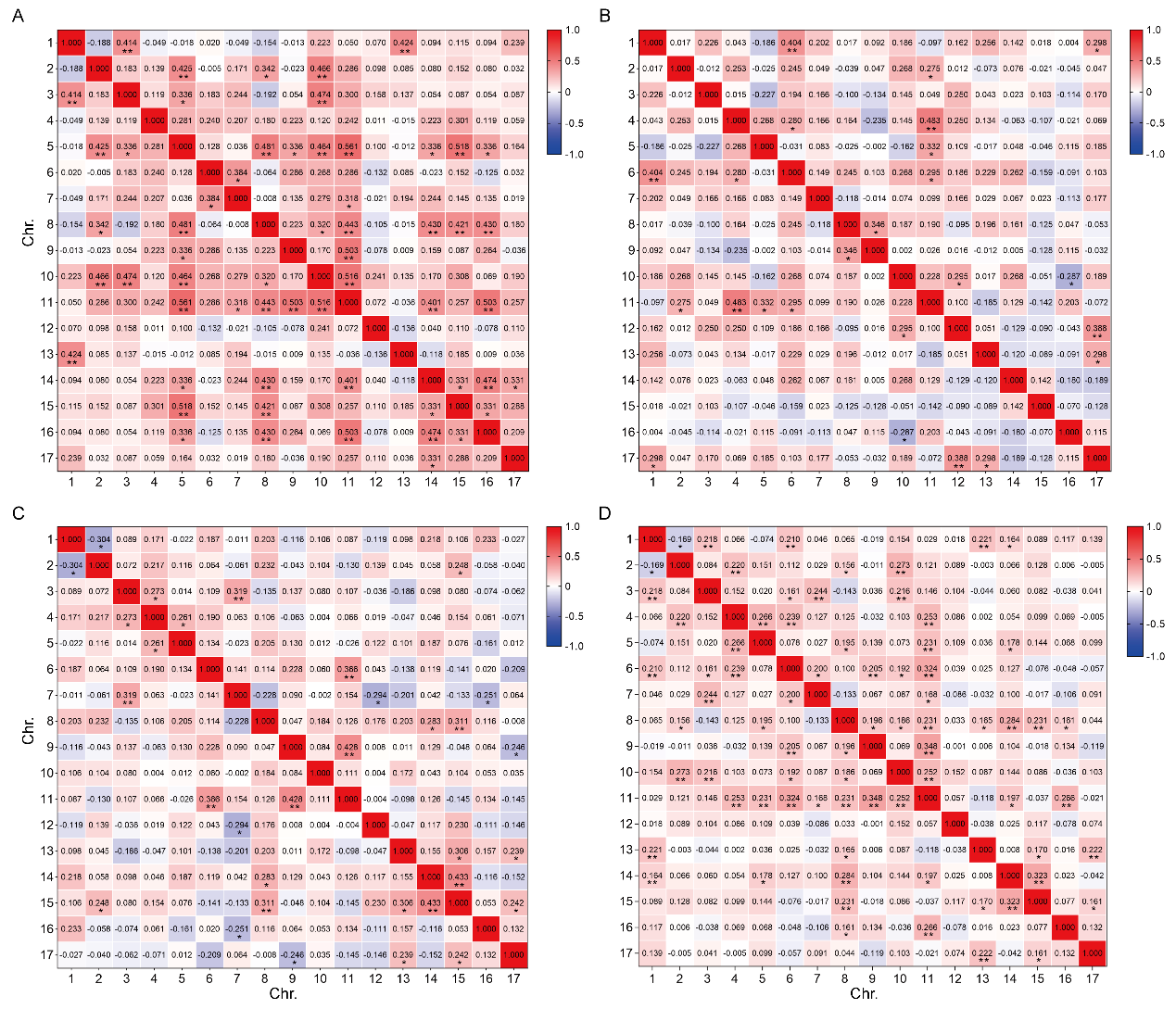


**Fig. S8** **The relationship between chromosomes to analyze the disomic gametes that simultaneously received from the triploid parent by Spearman's rank. A-D** Heatmaps depicting the correlation between chromosomes with two copies obtained from triploid parent using Spearman rank correlation analysis in the Q×H2, Q×H1, Q×Z, and the three crosses offspring. According to the value of Spearman correlation coefficient, the correlation strength can be divided into the following categories: completely unrelated (*r_h_* = 0); a very weak correlation (0 < *r_h_* < 0.20); a weak correlation (0.20 ≤ *r_h_* < 0.40); a moderate correlation (0.40 ≤ *r_h_* < 0.70); a strong correlation (0.70 ≤ *r_h_* < 0.90); a very strong correlation (0.90 ≤ *r_h_* < 1); Perfect correlation (*r_h_* = 1). “*” indicates significant difference (*p* < 0.05). “**” indicates highly significant difference (*p* < 0.01). The numbers in the heatmap represent the Spearman correlation coefficients. The 17 chromosomes of loquat appear on the left and at the bottom of the heatmap.


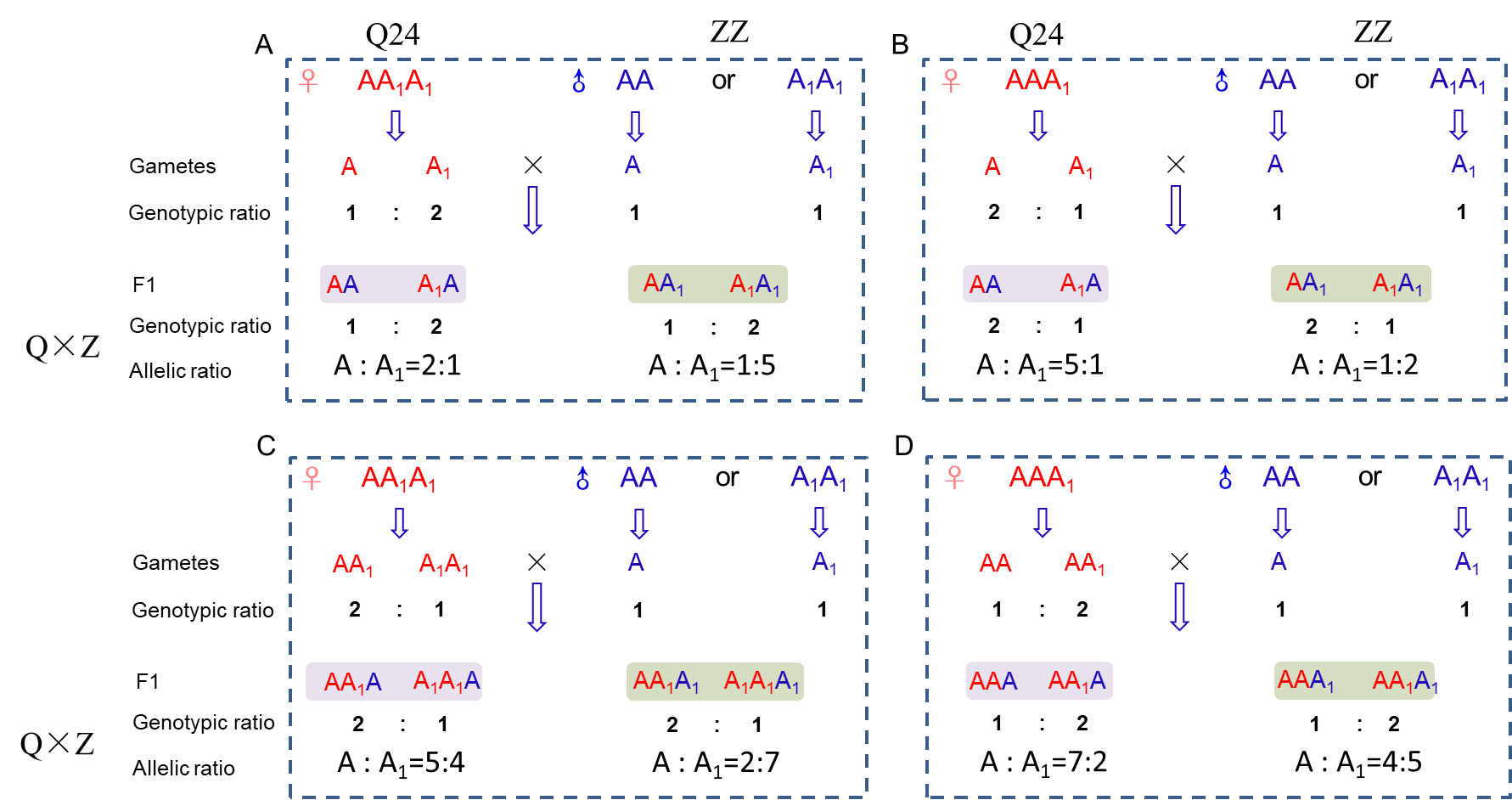


**Fig. S9 Expected inheriting efficiencies of alleles (AAA_1_ and AA_1_A_1_) of triploid loquat Q24, based on the Law of Segregation and Independent Assortment.** **A and B** respectively represent the inheriting efficiencies when triploid contribute only one allele. **C and D** inheriting efficiencies when triploid contribute two alleles.


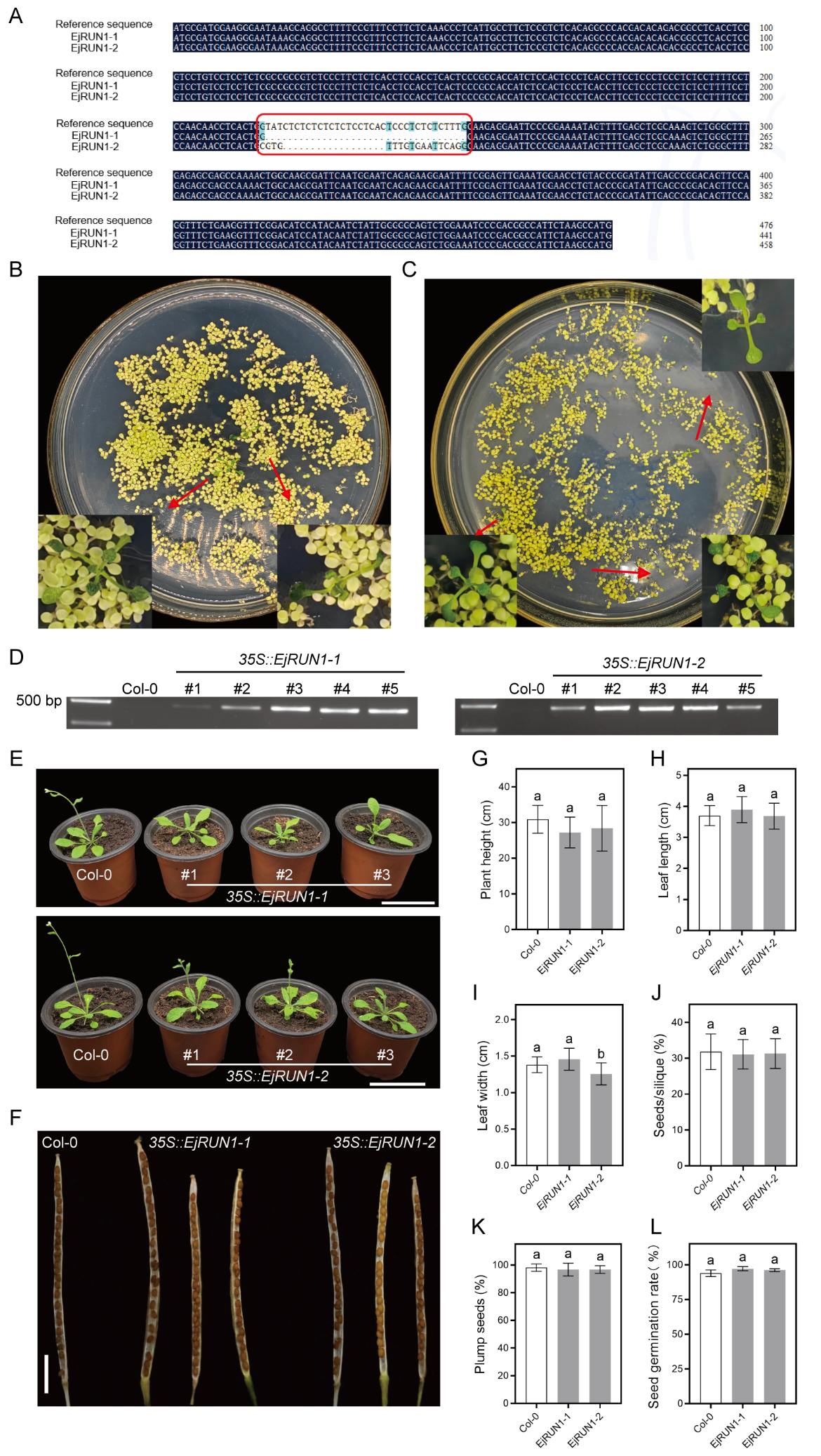


**Fig. S10 Identification and phenotypic observation of *A. thaliana* strains overexpressing *EjRUN1-1* and *EjRUN1-2*.** **A** Sequence alignment of *EjRUN1-1* and *EjRUN1-2*. The red box represents the region of variation encoding the exon. **B-C** respectively represents the screening of *EjRUN1-1* and *EjRUN1-2* transgenic *A. thaliana* lines by antibiotics. **D** Identification of *EjRUN1-1* and *EjRUN1-2* transgenic *A. thaliana* lines by PCR experiment. **E** Overexpression of *EjRUN1-1* and *EjRUN1-2* delayed *A. thaliana* flowering. **F** Silique phenotypes of transgenic and nontransgenic strains. **G-H** Main phenotypes of overexpressed plants: **G** Plant height. **H** Leaf length. **I** Leaf width. **J** Average number of seeds. **K** Number of plump seeds. **L** Seed germination rate. Error bars indicate means ± SEs.


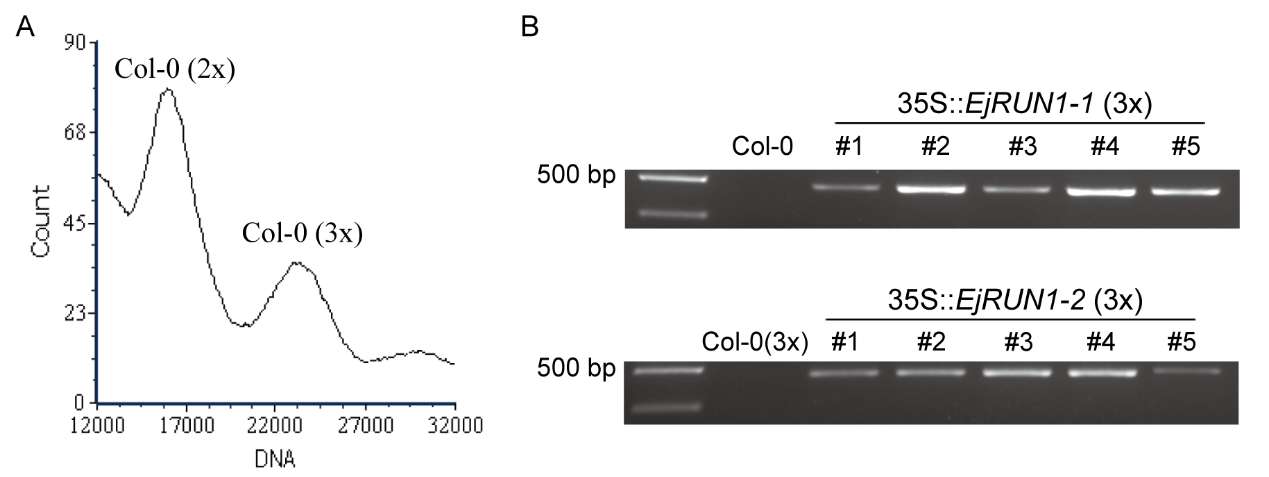


**Fig S11. Identification of triploid *A. thaliana* lines.** **A** Identification of triploid wild-type *A. thaliana* by flow cytometry. **B** Verification of *EjRUN1-1* and *EjRUN1-2* triploid transgenic *A. thaliana* lines by PCR experiment.


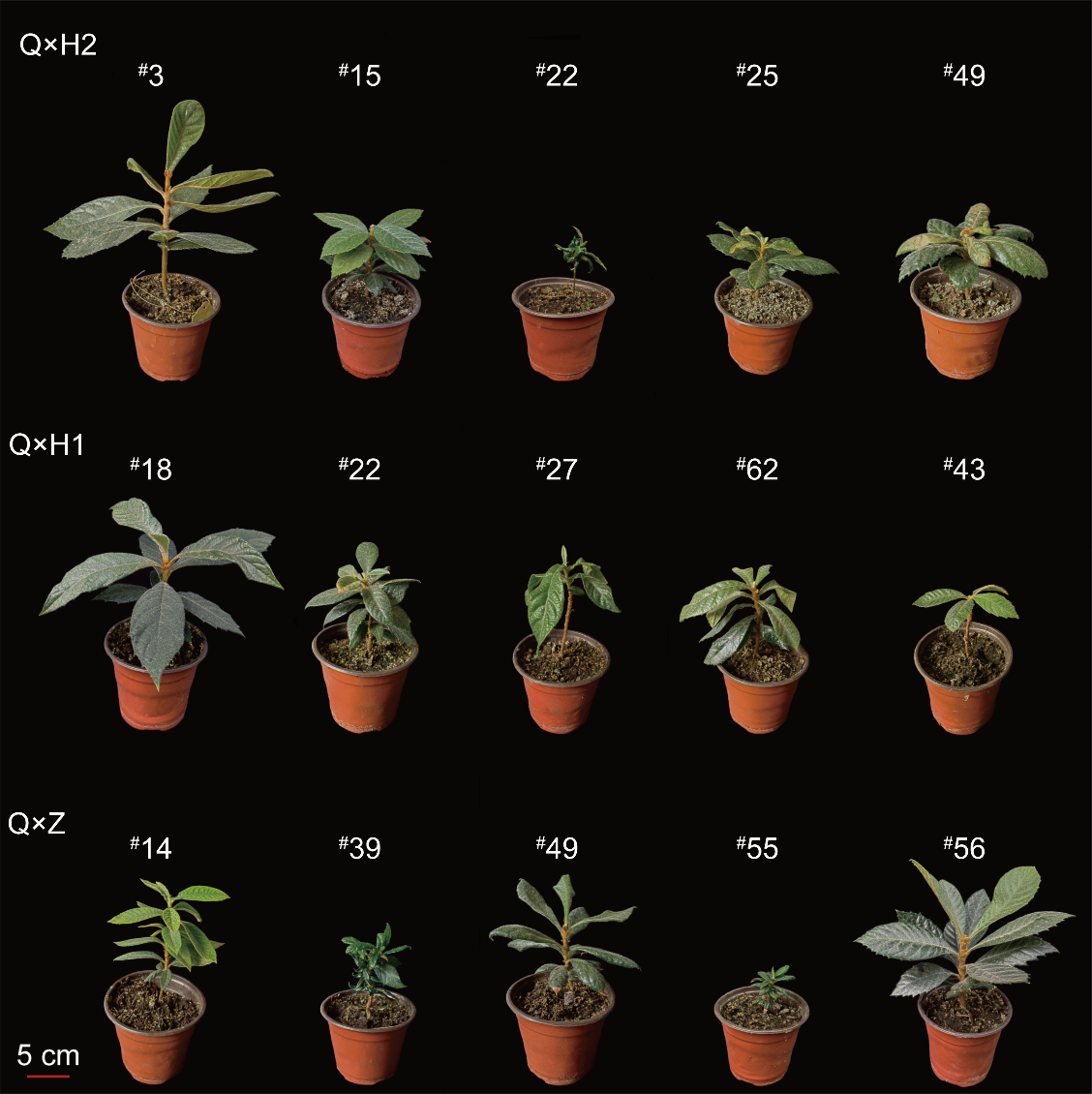


**Fig. S12 The aneuploid of loquats exhibits diverse phenotypes.** The directly above the image was the plant number. The upper left corner represents the hybrid combination number.


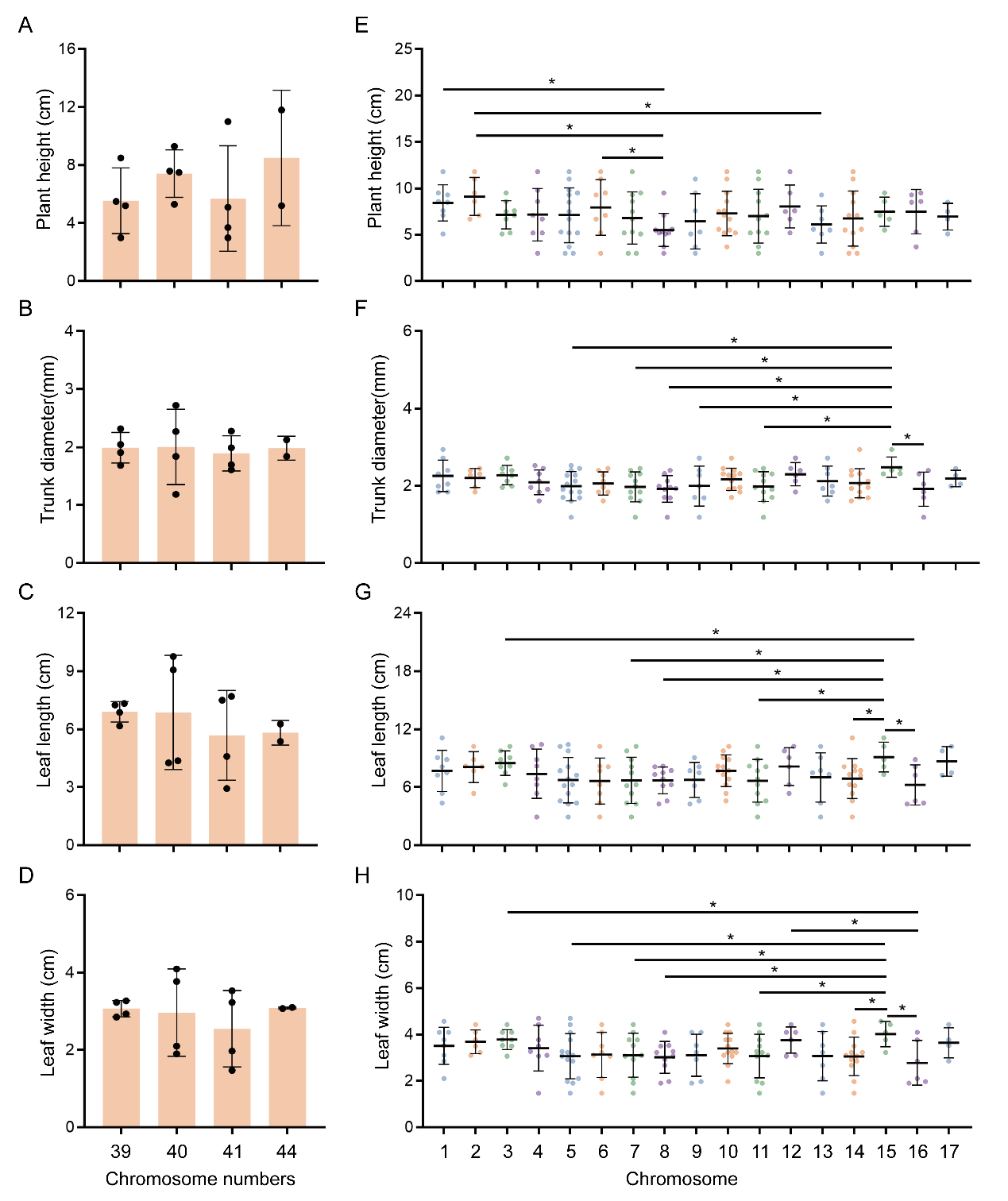


**Fig. S13 The impact of variation in chromosome number and dosage on the aneuploid phenotypes** **of Q×H1 offspring.** **A-D** indicated the effects of chromosome numbers variation in the plant height, trunk diameter, leaf length, and leaf width of Q×H1 cross offspring, respectively. The *x*-axis represents the chromosome numbers of loquat strains. **E-F** indicated the effects of chromosome dosage changes in the plant height, trunk diameter, leaf length, and leaf width of Q×H1 cross offspring, respectively. The *y*-axis represents the 17 chromosomes of loquat. Each dots represents a loquat strain. The one-way ANOVA test: *, *p* < 0.05.


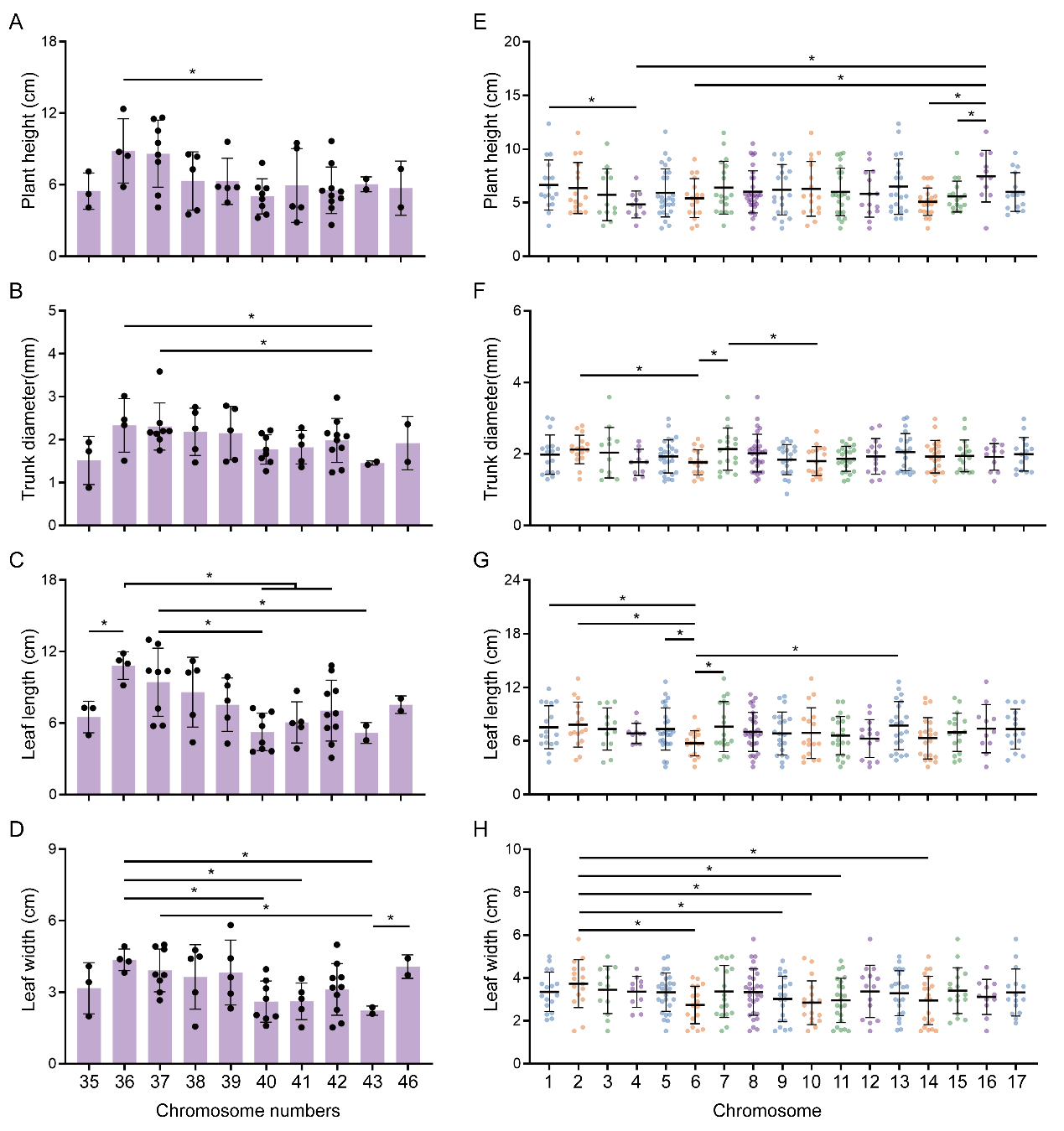


**Fig. S14** **The impact of variation in chromosome number and dosage on the aneuploid phenotypes of Q×Z offspring.** **A-D** indicated the effects of chromosome numbers variation in the plant height, trunk diameter, leaf length, and leaf width of Q×Z cross offspring, respectively. The *x*-axis represents the chromosome numbers of loquat strains. **E-F** indicated the effects of chromosome dosage changes in the plant height, trunk diameter, leaf length, and leaf width of Q×Z cross offspring, respectively. The *y*-axis represents the 17 chromosomes of loquat. Each dots represents a loquat strain. The one-way ANOVA test: *, *p* < 0.05.
