# Supplementary material for: Genome assembly of pomegranate highlights structural variations driving population differentiation and key loci underpinning cold adaption
Source: Hortic Res. 2025 Jan 21;12(5):uhaf022. doi: 10.1093/hr/uhaf022 (PMC11979328; doi:10.1093/hr/uhaf022)
Supplement: Web_Material_uhaf022 [file web_material_uhaf022.zip › Supplementary File - Tables.docx]

**Table S1 Primer information for 21 SSRs.**

| SSR name | Primer sequence (5’ to 3’) | Chr. | Product size (bp) | Annealing temperature (°C) |
| --- | --- | --- | --- | --- |
| MK1103 | F: TAGACAAACCCGGAAGATGC  R: CGTGATGGTGATGGTAGACG | 1 | 130 | 62.00 |
| MK2114 | F: TGTTCTTCCCCATGTTGGTT  R: GAAAGCCATCAGCTTTACGG | 1 | 138 | 62.00 |
| MK2376 | F: TCTGTGCCCATCTTCTTCTG  R: CACGATGTCGTAGCAAGGTTT | 1 | 136 | 62.00 |
| MK12200 | F: CTGCATCATGGTGGATCAGT  R: GCGCCTTAACCTCTCTCCTT | 2 | 128 | 62.00 |
| MK20023 | F: TTTTGCTTCGCACACTCTTG  R: CCCATGTAAGGGGAGCTACA | 3 | 167 | 62.00 |
| MK30145 | F: CGCATGAGGAATCGAAGTTT  R: CCTACTCTCCGCATGTTGGT | 4 | 149 | 62.00 |
| MK38418 | F: TTGACCAGGAGGGTTCTCTT  R: AAAAGACATCATCCGCAAGG | 5 | 124 | 62.00 |
| MK46485 | F: ACAGGGGATAAAACCGGAAC  R: ACACCCTGAGCAAATGGAAG | 6 | 147 | 62.00 |
| MK57627 | F: TGAAACCCCTGCAACAAAAT  R: TATCACGCACCATTTGAACC | 7 | 128 | 62.00 |
| MK63576 | F: TGGCATGATCTAAGCCCTTC  R: TTTGAAATCCAACCCTCCAG | 8 | 128 | 62.00 |
| MK68868 | F: TCGACCACGATCTAATGCAG  R: GACAACCACCTTCACGGAGT | 9 | 150 | 62.00 |
| MK77720 | F: CTTCTTGCCCAACACCACTT  R: GAACCCTTTGTCCCTCCTTC | 10 | 144 | 62.00 |
| MK82869 | F: TTGTCCACCGTTTCAAATCA  R: TTTGGTGCCATACCTCTTCC | 11 | 134 | 62.00 |
| MK83763 | F: TGCCATGAATACCAAACCAA  R: AAGGGCCACGATTTTCTCTT | 11 | 130 | 62.00 |
| MK93183 | F: AAGTTTGCCGAAACATGGAG  R: CGGCGAAGAGAGAGAGAGAA | 12 | 157 | 62.00 |
| MK102758 | F: GACTTGACCGGTTGGACACT  R: TCCCCAATTGATTTCAGAGC | 13 | 139 | 62.00 |
| MK105637 | F: GGACCGGGGTGTTACAGTATT  R: GACCTTACCTGTGCGTCCTC | 14 | 158 | 62.00 |
| MK112022 | F: AGGGCGTAACATCACAATCG  R: CGATGTAGTGCCTTTGGACA | 15 | 138 | 62.00 |
| MK119744 | F: AATGAATCCAAGGTAGCACGA  R: TGGTTTGAACATCGCATACA | 16 | 151 | 62.00 |
| MK127913 | F: AATGCTGCCTAGCAGTTGGT  R: CAAATGGGTGGAAATGCTCT | 17 | 130 | 62.00 |
| MK128081 | F: CCCCTTTGCTGTTCTTTATTTG  R: AAAATCCCCAAGAAGTTCCA | 17 | 125 | 62.00 |

Chr., chromosome; F, forward; R, reverse.

**Table S2** **ΔRn values of 21 pairs of SSR primers in four** **parental materials**

| SSR name | Chr. | Q24 | HB1 | HB2 | ZZ | Mean control |
| --- | --- | --- | --- | --- | --- | --- |
| MK1103 | 1 | 1.72±0.03 | 1.69±0.04 | 1.69±0.04 | 1.90±0.04 | 1.75±0.08 |
| MK2114 | 1 | 1.63±0.01 | 1.57±0.02 | 1.57±0.02 | 1.38±0.04 | 1.54±0.12 |
| MK2376 | 1 | 1.04±0.00 | 1.02±0.02 | 1.03±0.02 | 1.05±0.01 | 1.04±0.01 |
| MK12200 | 2 | 0.90±0.01 | 0.94±0.04 | 0.91±0.01 | 1.01±0.01 | 0.94±0.08 |
| MK20023 | 3 | 1.06±0.01 | 1.09±0.05 | 1.24±0.01 | 1.22±0.10 | 1.15±0.07 |
| MK30145 | 4 | 1.00±0.03 | 1.00±0.04 | 1.00±0.03 | 1.00±0.04 | 1.00±0.00 |
| MK38418 | 5 | 0.88±0.03 | 0.87±0.01 | 0.83±0.01 | 0.95±0.04 | 0.88±0.05 |
| MK46485 | 6 | 1.03±0.03 | 1.03±0.00 | 1.04±0.02 | 0.96±0.03 | 1.02±0.03 |
| MK57627 | 7 | 0.88±0.02 | 0.88±0.01 | 0.86±0.01 | 0.90±0.03 | 0.88±0.03 |
| MK63576 | 8 | 0.87±0.03 | 0.92±0.03 | 0.90±0.01 | 0.92±0.04 | 0.90±0.06 |
| MK68868 | 9 | 0.99±0.03 | 0.98±0.02 | 0.94±0.04 | 1.03±0.05 | 0.99±0.05 |
| MK77720 | 10 | 0.90±0.01 | 0.81±0.02 | 0.79±0.01 | 0.77±0.05 | 0.82±0.12 |
| MK82869 | 11 | 0.86±0.02 | 0.91±0.04 | 0.91±0.04 | 0.99±0.05 | 0.92±0.04 |
| MK83763 | 11 | 0.93±0.02 | 0.93±0.01 | 0.93±0.00 | 0.97±0.03 | 0.94±0.02 |
| MK93183 | 12 | 0.97±0.03 | 0.91±0.03 | 0.93±0.04 | 0.87±0.04 | 0.92±0.03 |
| MK102758 | 13 | 0.96±0.03 | 0.90±0.02 | 0.93±0.02 | 0.98±0.06 | 0.94±0.07 |
| MK105637 | 14 | 1.78±0.01 | 1.72±0.05 | 1.71±0.04 | 1.43±0.04 | 1.66±0.14 |
| MK112022 | 15 | 0.77±0.02 | 0.86±0.02 | 0.85±0.01 | 0.93±0.01 | 0.85±0.07 |
| MK119744 | 16 | 0.87±0.00 | 0.98±0.04 | 1.02±0.02 | 0.93±0.03 | 0.95±0.05 |
| MK127913 | 17 | 1.42±0.05 | 1.50±0.05 | 1.62±0.01 | 1.46±0.01 | 1.50±0.07 |
| MK128081 | 17 | 0.97±0.03 | 0.99±0.02 | 0.98±0.04 | 0.81±0.04 | 0.94±0.07 |

Note, that the SSR-qPCR results are expressed as the means ± standard deviations; Chr., chromosome.

**Table S3** The coefficient variation of four phenotypes of Q×H2 offspring.

| Cross types | Ploidy | Plant height | Trunk diameter | Leaf length | Leaf width |
| --- | --- | --- | --- | --- | --- |
| Q×H2 | euploid group | 28.98% | 26.92% | 30.13% | 25.13% |
| Q×H2 | aneuploid group | 54.88% | 40.37% | 50.03% | 40.61% |

Coefficient variation= (standard deviation/ the average value) × 100%.

**Table S4** Information on the sequencing results.

| Sample | HQ reads | HQ reads(%) | HQ data(bp) | HQ data(%) | Tatal reads | Mapped reads | Mapping rate |
| --- | --- | --- | --- | --- | --- | --- | --- |
| Q11-4 | 18,065,836 | 93.62% | 2,547,712,663 | 91.68% | 18,157,566 | 18,127,013 | 99.83% |
| H324-10 | 15,702,210 | 93.96% | 2,217,677,172 | 92.15% | 15,768,266 | 15,723,569 | 99.72% |
| H39 | 15,512,034 | 94.05% | 2,191,207,251 | 92.26% | 15,575,816 | 14,706,190 | 94.42% |
| Q11-5 | 17,219,178 | 93.55% | 2,429,425,684 | 91.66% | 17,333,125 | 17,309,018 | 99.86% |
| Q27-11 | 15,908,582 | 94.07% | 2,245,664,630 | 92.21% | 15,975,406 | 15,853,073 | 99.23% |
| Q27-1 | 13,158,306 | 93.53% | 1,855,285,464 | 91.58% | 13,238,440 | 13,218,212 | 99.85% |
| Q×H1-10 | 15,032,966 | 96.91% | 2,140,730,667 | 95.84% | 15,143,851 | 14,982,146 | 98.93% |
| Q×H1-11 | 16,026,856 | 96.99% | 2,282,556,650 | 95.92% | 16,135,496 | 15,987,749 | 99.08% |
| Q×H1-17 | 16,311,756 | 93.55% | 2,300,371,816 | 91.62% | 16,388,633 | 16,362,940 | 99.84% |
| Q×H1-23 | 12,396,316 | 93.63% | 1,748,802,175 | 91.72% | 12,473,284 | 12,456,528 | 99.87% |
| Q×H1-24 | 19,314,224 | 97.05% | 2,751,640,908 | 96.02% | 19,426,474 | 17,751,688 | 91.38% |
| Q×H1-30 | 29,826,844 | 96.13% | 4,230,604,536 | 94.69% | 30,042,594 | 29,596,111 | 98.51% |
| Q×H1-42 | 13,510,214 | 93.87% | 1,906,479,579 | 91.99% | 13,593,346 | 13,574,271 | 99.86% |
| Q×H1-47 | 16,790,240 | 96.96% | 2,390,896,553 | 95.88% | 16,896,816 | 16,682,307 | 98.73% |
| Q×H1-52 | 17,675,496 | 94.29% | 2,497,476,720 | 92.52% | 17,761,213 | 17,736,013 | 99.86% |
| Q×H1-5 | 14,155,462 | 97.19% | 2,017,468,918 | 96.19% | 14,237,674 | 13,845,970 | 97.25% |
| Q×H2-13 | 15,985,460 | 96.99% | 2,275,257,173 | 95.87% | 16,082,163 | 15,801,107 | 98.25% |
| Q×H2-14 | 19,137,762 | 93.77% | 2,700,274,189 | 91.88% | 19,226,802 | 19,201,521 | 99.87% |
| Q×H2-16 | 15,319,404 | 93.94% | 2,163,429,044 | 92.13% | 15,403,575 | 14,471,541 | 93.95% |
| Q×H2-23 | 12,176,400 | 93.11% | 1,714,625,163 | 91.05% | 12,272,740 | 12,254,598 | 99.85% |
| Q×H2-29 | 15,714,140 | 96.71% | 2,234,768,215 | 95.51% | 15,803,795 | 15,238,457 | 96.42% |
| Q×H2-3 | 16,293,380 | 94.33% | 2,302,416,372 | 92.57% | 16,364,749 | 16,340,782 | 99.85% |
| Q×H2-6 | 15,000,298 | 96.97% | 2,136,963,919 | 95.94% | 15,109,368 | 14,694,826 | 97.26% |
| Q×H2-7 | 13,349,254 | 92.93% | 1,879,393,823 | 90.85% | 13,438,662 | 13,418,234 | 99.85% |
| Q×H2-9 | 13,080,226 | 94.10% | 1,847,356,272 | 92.29% | 13,173,057 | 13,151,378 | 99.84% |
| Q×Z-11 | 15,177,670 | 93.70% | 2,141,323,530 | 91.80% | 15,248,390 | 15,225,155 | 99.85% |
| Q×Z-16 | 12,991,422 | 93.15% | 1,830,468,882 | 91.14% | 13,109,705 | 13,082,088 | 99.79% |
| Q×Z-17 | 16,517,798 | 94.47% | 2,335,162,938 | 92.75% | 16,583,359 | 16,559,827 | 99.86% |
| Q×Z-25 | 16,100,600 | 93.23% | 2,268,224,839 | 91.21% | 16,173,564 | 16,087,609 | 99.47% |
| Q×Z-37 | 14,517,056 | 94.42% | 2,051,938,805 | 92.68% | 14,581,819 | 14,561,504 | 99.86% |
| Q×Z-50 | 12,417,172 | 94.06% | 1,754,548,646 | 92.30% | 12,567,618 | 12,549,813 | 99.86% |
| Q×Z-51 | 15,553,914 | 93.88% | 2,195,296,886 | 92.02% | 15,654,805 | 15,636,100 | 99.88% |
| Q×Z-60 | 19,109,392 | 94.31% | 2,701,076,242 | 92.57% | 19,212,746 | 19,183,959 | 99.85% |
| Q×Z-8 | 17,934,060 | 93.95% | 2,532,874,816 | 92.14% | 18,010,356 | 17,986,388 | 99.87% |
